# Bionanofactory for green synthesis of collagen nanoparticles, characterization, optimization, in-vitro and in-vivo anticancer activities

**DOI:** 10.1038/s41598-024-56064-8

**Published:** 2024-03-15

**Authors:** Asmaa A. El-Sawah, Noura El-Ahmady El-Naggar, Heba E. Eldegla, Hoda M. Soliman

**Affiliations:** 1 Botany Department, Faculty of Science, Mansoura University, Mansoura, Egypt; 2 Department of Bioprocess Development, Genetic Engineering and Biotechnology Research Institute, City of Scientific Research and Technological Applications (SRTA-City), New Borg El-Arab City, 21934 Alexandria Egypt; 3 Medical Microbiology and Immunology Department, Faculty of Medicine, Mansoura University, Mansoura, Egypt

**Keywords:** Collagen nanoparticles, Biosynthesis, *Streptomyces plicatus*, Characterization, Optimization, FCCD, Antioxidant, Anticancer, Drug loading, Nanoparticles, Applied microbiology

## Abstract

Collagen nanoparticles (collagen-NPs) are promising biological polymer nanoparticles due to their exceptional biodegradability and biocompatibility. Collagen-NPs were bio-fabricated from pure marine collagen using the cell-free supernatant of a newly isolated strain, *Streptomyces* sp. strain NEAA-3. *Streptomyces* sp. strain NEAA-3 was identified as *Streptomyces plicatus* strain NEAA-3 based on its cultural, morphological, physiological properties and 16S rRNA sequence analysis. The sequence data has been deposited under accession number OR501412.1 in the GenBank database. The face-centered central composite design (FCCD) was used to improve collagen-NPs biosynthesis. The maximum yield of collagen-NPs was 9.33 mg/mL with a collagen concentration of 10 mg/mL, an initial pH of 7, an incubation time of 72 h, and a temperature of 35 °C. Using the desirability function approach, the collagen-NPs biosynthesis obtained after FCCD optimization (9.53 mg/mL) was 3.92 times more than the collagen-NPs biosynthesis obtained before optimization process (2.43 mg/mL). The TEM analysis of collagen-NPs revealed hollow sphere nanoscale particles with an average diameter of 33.15 ± 10.02 nm. FTIR spectra confirmed the functional groups of the collagen, collagen-NPs and the cell-free supernatant that are essential for the efficient capping of collagen-NPs. The biosynthesized collagen-NPs exhibited antioxidant activity and anticancer activity against HeP-G2, MCF-7 and HCT116 cell lines. Collagen-NPs assessed as an effective drug loading carrier with methotrexate (MTX), a chemotherapeutic agent. The TEM analysis revealed that the average size of MTX-loaded collagen-NPs was 35.4 ± 8.9 nm. The percentages of drug loading (DL%) and encapsulation efficiency (EE%) were respectively 22.67 and 45.81%.

## Introduction

In recent years, nanoparticles have attracted a lot of attention due their unique characteristics. There are several types of nanoparticles, including metal nanoparticles, carbon-based nanoparticles, ceramic nanoparticles, lipid-derived nanoparticles, and polymeric nanoparticles. Polymeric nanoparticles are very small, granular, and colloidal particles with a variety of shapes and structures. There are many different types of natural polymeric nanoparticles, such as those made of collagen, soy, keratin, silk, and elastin. The advantageous characteristics of these particles are their bioavailability, biodegradability, and low costs^1^.

Collagen, the most abundant protein in the human body, is found in the bones, muscles, skin, and other connective tissues. The well-known protein collagen has been utilized extensively in medical applications, including the development of microspheres and microneedles for the administration of drugs^2^, the production of protein delivery tablets and pellets^3^, for the therapy of cancer^4^, the production of gels and the incorporation of liposomes and medications for long-term administration of medicines^5^, and collagen shields in eye disease^6^. Collagen-NPs can disperse in water to produce colloidal solutions because of their small size, large surface area, and absorption capacity. Additionally, collagen-NPs enhance cell retention, can be quickly sterilized, maintain their shape under heat, and minimize the dangers of dangerous byproducts created during breakdown^7^. Collagen-NPs are more advantageous than other naturally occurring and synthetic polymeric NPs as a result of their outstanding biodegradability and biocompatibility, high contact surface, minimal antigenicity, lowered toxicity, and elevated cationic-charge density potential caused by their significant abundance of amino groups. This continues to be true even in the lack of target compound-induced surface change^8^.

Traditionally, Collagen-NPs are synthesized using physical and chemical methods, both of which have several drawbacks. The use of extremely hazardous chemicals, environmental pollution, and carcinogenic solvents are the principal drawbacks of chemical methods that restrict their application in the clinical setting. The use of physical methods requires expensive equipments and an excessive amount of energy. TEM analysis revealed that the chemically synthesised collagen-NPs ranged in size from 50 to 500 nm by the self-assembly method^9^. After utilizing 4,4-azobis (4-cyanovaleric acid) and 3-acrylamidophenylboronic acid for synthesis, the TEM analysis revealed that the nanoparticles were spherical, evenly distributed, and had an average size of 81.3 nm. The diameter of the nanoparticles was around 79 nm^10^. By using poly diethylene glycol methyl ether methacrylate, the produced collagen-NPs recorded average diameter was about 100 nm^11^. The smaller size of nanoparticles is advantageous especially in biomedical applications. To synthesis of smaller size collagen-NPs for biomedical applications, there is a growing demand to develop techniques that are highly reliable, inexpensive, do not require the use of toxic substances, and eco-friendly process.

The green synthesis of nanoparticles was accomplished using biological resources as a potential nano-factories, such as microorganisms including bacteria, actinomycetes, algae or fungi are recognized as potential nano-factories for green synthesis of a variety of nanoparticles^12–15^. As well as or plant extracts^16–18^, algal pigments^19,20^, algal derived soluble polysaccharides^21^ and fungi^22^. Due to the fact that they function as bio reducers, capping/stabilizing agents, or both, microorganisms or their protein extracts can be utilised to green synthesise nanoparticles in an environmentally sustainable manner, without the need for external chemicals^23^.

Actinomycetes are a diverse microbial group recognized for producing antibiotics and other essential metabolites such as vitamins, antitumor agents, enzyme inhibitors, antibiotics, and enzymes. *Streptomyces* is the largest and most important genus in the order actinomycetales, and there are many species of *Streptomyces* in both terrestrial and aquatic environments. Insecticides, antiparasitic medications, immunostimulatory, immunosuppressive, antioxidative, anticancer, and other very useful pharmaceutical compounds with a wide range of medical and agricultural uses are also produced by *Streptomyces*^24–27^.

The goals of this study were to biosynthesize ultrafine collagen-NPs using eco-friendly approach, to identify a newly isolated strain, *Streptomyces* sp. strain NEAA-3 that can biosynthesize collagen-NPs, use the cell-free supernatant of *Streptomyces plicatus* strain NEAA-3 for biosynthesis of collagen-NPs, to determine the optimum collagen concentration, pH, temperature, and incubation time for maximum biosynthesis of collagen-NPs using a face-centered central composite design, to evaluate the collagen-NPs' antioxidant, and anticancer activity on the HeP-G2, MCF-7, and HCT116 cell lines, to evaluate collagen-NPs' ability to supress tumor growth in mice bearing Ehrlich ascites carcinoma (EAC) and its potential to drug loading for methotrexate (MTX).

## Materials and methods

**Figure 1:**
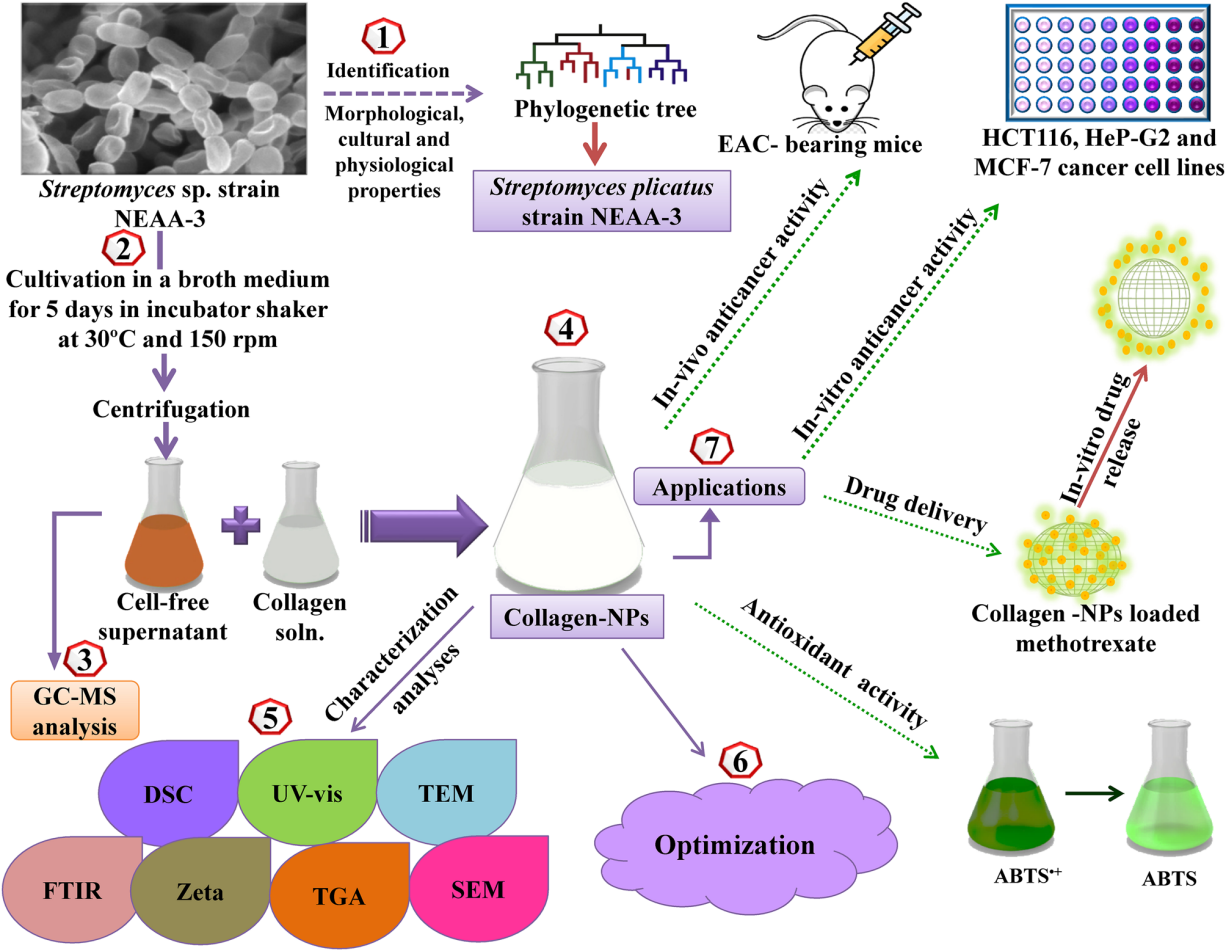
Schematic diagram of the research process in this study.

A schematic representation of the research design framework of this study has been provided in Fig. 1.

### Microorganism and cultivation conditions

In this investigation, *Streptomyces* sp. strain NEAA-3 was isolated and kindly provided by Prof. Noura El-Ahmady El-Naggar, 2nd author. *Streptomyces* sp. strain NEAA-3 was cultured on Petri dishs containing starch-nitrate agar medium which had the following components (g/L) agar, 20; soluble starch, 20; KNO_3_, 2; NaCl, 0.5; K_2_HPO_4_, 1; FeSO_4_·7H_2_O, 0.01; and 3; CaCO_3_. The inoculated Petri dishs were then incubated for seven days at 30 °C. *Streptomyces* sp. strain NEAA-3 was stored as spore suspensions in a 20% (v/v) glycerol solution at − 20 °C for future investigation.

### Morphological and cultural properties

*Streptomyces* sp. strain NEAA-3 's spore surface ornamentation and spore chain morphology were investigated by TEM (JEOL JSM 6510) after 14 days of incubation at 30 °C on a medium of starch nitrate agar. The pigmentation of substrate mycelium, color of aerial mycelium, and diffusible pigments production also were studied following the methods of Shirling and Gottlieb^28^ on ISP 1 (tryptone-yeast extract agar), ISP 2 (yeast-malt extract agar), ISP 3 (oatmeal agar), ISP 4 (inorganic salt starch agar), ISP 5 (glycerol- asparagine agar), ISP 6 (peptone-yeast extract iron agar), ISP 7 (tyrosine agar); within days, all plates were incubated at 30 °C.

### physiological properties

*Streptomyces* sp. strain NEAA-3 was investigated for the utilization of different carbon sources, melanin production, NaCl tolerance, milk coagulation and gelatin liquefaction using the methods described by Shirling and Gottlieb^28^. To examine the strain's ability to produce α-amylase, it was streaked onto a starch nitrate agar medium plate with 2% soluble starch and grown for 7 days at 28 °C. After the incubation, the plate was stained using Gram's iodine solution^29^.

### Phylogenetic analysis, 16S rRNA sequencing and sequence alignment

16S rRNA of *Streptomyces* sp. strain NEAA-3 was extracted based on the method of Sambrook et al.^30^. By using PCR, 16S rRNA was amplified using El-Naggar et al.^31^ technique. The GenBank database has assigned accession number OR501412.1 to the resulting sequences. Using the BLAST tool (https://blast.ncbi.nlm.nih.gov/Blast.cgi;^32^), the 16S rRNA gene sequence (partial) of strain NEAA-3 was matched with other related species of the *Streptomyces* genus using the corresponding 16S rRNA sequences collected from the GenBank, EMBL, DDBJ, and PDB databases. A phylogenetic tree was generated using MEGA 11 software tool^33^.

### Cell-free supernatant preparation

*Streptomyces plicatus* strain NEAA-3 was cultured on starch nitrate agar medium. Six discs with a diameter of 9 mm were taken from the culture that had been prepared previously and inoculated into 250 mL Erlenmeyer flasks with 100 mL of the medium consists of the following components: Soluble starch, 20; MgSO_4_, 0.5; KNO_3_, 1; NaCl, 0.5; K_2_HPO_4_, 0.5; FeSO_4_·7H_2_O, 0.1 and 0.3 yeast extract; 1 L of distilled H_2_O. According to Mohamedin et al.^14^, flasks were incubated for five days at 30 °C and 150 rpm in an incubator shaker. The mycelium was subsequently separated from the cell-free supernatant using centrifugation for 15 min at 4 °C. The cell-free supernatant was lyophilized before being characterized by GC–MS analysis.

### Extracellular biosynthesis of Collagen-NPs

For the biosynthesis of collagen-NPs, 9 mL of freshly prepared cell-free supernatant (pH was adjusted to 7) was mixed with 1 mL of 10 mg/mL of pure marine collagen type I solution (MM Ingredients Ltd, UK) and incubated at 35 °C for 48 h. The appearance of white turbidity indicates the biosynthesis of collagen-NPs. After the biosynthesis of collagen-NPs using the cell-free supernatant of *Streptomyces plicatus* strain NEAA-3, the collagen-NPs was centrifuged at 13,000×*g* for 10 min, and then the supernatant was discarded. The pellets (collagen-NPs) were washed three times with distilled water for complete elimination of the cell-free supernatant and then re-dispersed by distilled water. Freeze-dried collagen-NPs were used for characterisation and application.

### Characterization of collagen-NPs

The spectroscopic investigation was performed on the biosynthesized collagen-NPs, and the UV–vis absorbance was measured between 200 and 800 nm using the ATI Unicam 5625 UV/VIS Vision Software V3.20. Transmission electron microscopy (TEM) was used to detect the morphology and size of the collagen-NPs using JEOL-JEM-100 CXII instrument, and scanning electron microscopy (SEM, JEOL JSM 6510 lv) was used to characterize the freeze-dried collagen-NPs. The freeze-dried collagen-NPs' elemental composition was identified by Energy-dispersive X-ray (EDX) using Oxford X-Max 20. Fourier-Transform Infrared (FTIR, thermo Scientific Nicolet iS10) was employed to determine the functional groups included in the freeze-dried collagen-NPs. The X-ray diffraction analysis (XRD) spectra of collagen powder and collagen-NPs were recorded using a Bruker D2 PHASER 2nd Gen X-ray diffractometer. The samples were exposed to monochromatic Cu/K radiations (= 1.5405) with diffraction angles varying from 10 to 80° at a scanning rate of 2 min^−1^. Zeta potential analysis was used to determine the net surface charge of the biosynthesized collagen-NPs using the Zeta sizer nano ZS90 (Malvern Instruments Ltd. in the United Kingdom).

The thermal decomposition behavior of collagen and collagen-NPs was examined using DSC (differential scanning calorimetry). The scans were conducted at a variety of temperatures, from ambient temperature to 500 °C. The graph depicted heat flow against temperature. TGA (Thermogravimetric analysis) of collagen and collagen-NPs were completed using a thermo-analyzer of type 50-H. Both samples were subjected to TGA investigation at temperatures ranging from room temperature to 800 °C in 10 °C min^−1^ increments. In a nitrogen atmosphere with a flow rate of 30 mL/min, samples of freeze-drying collagen and collagen-NPs weighing approximately 2.1 mg and 1.7 mg, respectively, were analyzed. The graph depicted the relationship between weight loss and temperature.

### Optimization of collagen-NPs biosynthesis by Face central composite design (FCCD)

The FCCD, with thirty experiments and three replicates, was used to study the effects of four variables on the biosynthesis of collagen-NPs, including collagen concentration (X_1_), pH level (X_2_), temperature (X_3_), and incubation period (X_4_). Each design variable has been examined at three different levels (− 1, 0, and 1). The central values (zero level) for the experimental design were 10 mg/mL of collagen solution, 48 h of incubation time, pH level 7, and 35 °C. The second polynomial regression equation was used to fit the experiment data:

1 $$Y = \beta_{0} + \sum\limits_{i} {\beta_{i} X_{i} + \sum\limits_{ii} {\beta_{ii} X_{i}^{2} } } + \sum\limits_{ij} {\beta_{ij} X_{i} X_{j} }$$

where, Y is the predicted collagen-NPs and *β*_i_, *β*_ii_, *β*_ij_ are the linear, quadratic, and interaction terms, respectively. *β*_0_ is a constant. *X*_*i*_ and *X*_j_ are the coded levels symbolized as X_1_, X_2_, X_3_ and X_4_.

### Statistical analysis

Applying Design Expert 12 software for Windows (version 12; Stat-Ease Inc., USA) (https://www.statease.com/software/design-expert/), optimization and experimental data analysis were carried out. By using Version 8.0, StatSoft Inc., Tulsa, USA, STATISTICA software, the 3D surface graph of two different variables was plotted against the production of collagen-NPs (https://www.statsoft.de/de/software/statistica).

#### ABTS^+^ antioxidant activity test

3 mL of MnO_2_ (25 mg/mL) solution and 2 mL of (60 M) ABTS (2, 2′-Azino-bis (3-ethylbenzthiazoline-6-sulfonic acid) solution have been utilized for the detection of the antioxidant activity of collagen-NPs. Both had been pre-prepared in 5 mL of PBS (pH 7, 0.1 M). A green–blue solution (ABTS^+^ solution) was created by shaking, centrifuging, and filtering the mixture. The absorbance (A control) was then adjusted to a value of roughly 0.5 at 734 nm. Once the combination has sat for 30 min at room temperature, combine 1 mL of the ABTS^+^ solution with 1 mL of collagen-NPs (500 µg/mL), and use a microplate reader to measure the absorbance at 734 nm (A test). The same method was carried out to compare native collagen powder. The inhibition percentage was estimated as follows: (A_control_ − A_test_)/A_control_ × 100. Inhibition was utilized for expressing the absorbance (A_test_), which connected to the reduction of color intensity.

### In vitro anticancer activity of collagen-NPs on cancer and normal cell lines

In vitro anticancer activity of collagen-NPs was investigated against normal and cancer cell lines by utilizing a 3-(4, 5-Dimethylthiazol-2yl)-2, 5-diphenyl tetrazolium bromide (MTT) assay (colorimetric method). Cell lines for human lung fibroblast (WISH), human amnion (WISH), human colorectal carcinoma (HCT116), human liver cancer (HeP-G2), and mammary gland breast cancer (MCF-7) were obtained from the American Type Culture Collection (ATCC) through the Holding Corporation for Biological Products and Vaccines (VACSERA) in Cairo, Egypt. The appearance of formazan's purple color is directly proportional to the number of living cells because of the presence of NAD-dependent mitochondrial dehydrogenase in viable cells^34^. The cells were grown in an incubator at 37 °C with 5% CO_2_ using RPMI-1640 media with 10% fetal bovine serum, 100 units/mL penicillin, and 100 µg/mL streptomycin antibiotics. For single-cell suspensions, trypsin-ethylene diamine tetra acetic acid (EDTA) has been used to separate monolayer cells. A hemocytometer has been used to calculate the number of viable cells. In 96-well plates, 10,000 cells/well were seeded using 100 µL of cell suspensions. The cells were then incubated for 48 h at 37 °C with 5% CO_2_, 100% relative humidity, and 95% air to help the cells adhere to the bottoms of the wells. Collagen-NPs were employed to treat the cells at a range of concentrations (1.56, 3.125, 6.52, 12.5, 25, 50, and 100 µg/mL), and collagen-NPs concentrations were first passed through a 0.45-m filter syringe. Doxorubicin (DOX) was used as the reference. It was used after 24 h of incubation at 37 °C with 5% CO_2_ and 100% relative humidity. The study has been replicated to ensure the accuracy of the findings. 20 µL of the yellow MTT solution and 5 mg/mL of phosphate-buffered saline were administered to each well. The plates were incubated for MTT reduction for 4 h at 37 °C. Finally, 100 µL of DMSO was added to the generated purple formazan crystals. An EXL 800 plate reader was used to measure the absorbance at 570 nm.

The percentage of cytotoxicity has been determined by:

2 $${\text{Viability }}\% \, = \, \left( {{\text{Test OD}}/{\text{Control OD}}} \right) \, \times { 1}00$$

3 $${\text{Cytotoxicity }}\% \, = { 1}00 \, - {\text{Viability}}\%$$

### In vivo apoptosis of EAC by Collagen-NPs

The effect of collagen-NPs on the apoptosis of Ehrlich solid tumors has been investigated in vivo in order to determine whether or not collagen-NPs are able to induce apoptosis. The EAC cell line was chosen as it is one of the most prevalent tumors. Furthermore, EAC is classified as an undifferentiated carcinoma that is originally hyperdiploid, has a high transplantable capacity, does not regress, proliferates rapidly, and has a shorter life span. EAC is similar to human tumors in that it grows quickly and is undifferentiated, making it the most susceptible to chemotherapy^35^.

Adult Swiss female albino mice were obtained from the Institute of Theodore Bilharz Research in Giza, Egypt, weighing 25–30 g. They were kept and housed in standard-sized polycarbonate cages at the Faculty of Pharmacy, Mansoura University, Mansoura, Egypt, with unlimited access to food and water at all times. The animals were kept in laboratories at 26 ± 1 °C, 12-h light/dark cycle, 25 °C, and a relative humidity of 20%.

All mice were initially subcutaneously inoculated with 5 × 10^5^ EAC cells from Cairo University's National Cancer Institute (NCI), Cairo, Egypt, to produce solid tumors. Before the initiation of the treatment, volumes of solid tumors were roughly 50–100 mm^3^ (day 0) after five days of inoculation. The mice were then randomly divided into six groups. Each group included Swiss female albino mice (aged 9–10 weeks; 25–30 g; n = 5). Only EAC-bearing mice were in Group I, which served as the control. In Group II, EAC-bearing mice received a collagen injection in the tumor (2 mg/kg/1 day); in Group III, EAC-bearing mice received a doxorubicin (Dox) injection alone (2 mg/kg/1 day); in Group IV, EAC-bearing mice received a collagen-NPs alone (2 mg/kg/1 day); in Group V, EAC-bearing mice received a collagen in combination with DOX; and EAC-bearing mice received a collagen-NPs in combination with DOX. The same study design has been used in many previous studies as in^36–38^. Although the sample size was not high (n = 5) in each group, many previous researches used the same sample size as in^36,39,40^.

Tumor volumes were assessed after collagen-NPs and DOX inoculation on the fifth day after tumor formation and subsequently every 5 days for a total of 20 days. After completing the trial and treatment (day 21), mice were sacrificed by cervical dislocated under anesthesia according to the weight of the mouse with its tumor using approximately 1.4 mg thiopental sodium (40 mg/kg) and tumor lumps were taken out, weighed, and preserved in buffered formalin solution for histological analysis. The tumor's volume^38^ can be calculated with Vernier calipers using the following formula:

4 $${\text{V}} = \, \left( {{\text{L}} \times {\text{S}}^{{2}} } \right) \, \times \, 0.{5}$$

where, V is the volume of the tumor, L is the diameter of the longest tumor and S is the diameter of the shortest vertical tumor. According to the study of Schirner et al.^40^, the effectiveness of the anti-tumor was calculated as:

5 $${\text{Growth inhibition }}\left( \% \right){ } = { }100{ }{-}{ }\left( {{ }\frac{{{\Delta T}}}{{{\Delta C}}}{ } \times 100} \right)$$

where ΔT is the tumor volume change average in the treatment group and ΔC is the tumor volume change average in the control EAC-bearing mice.

Hematoxylin and eosin were used to stain the micrometer-sized slices, which were then viewed under a light microscope.

### Drug loading and encapsulation efficiency

10 mL of 5 mg/mL methotrexate (MTX) (Hikma Specialized Pharmaceuticals, Badr City, Cairo, Egypt) was added to 100 mg of collagen-NPs in order to assess the drug loading (DL%) and encapsulation efficiency (EE%). The mixture was stirred for 6 h at 25 °C. The precipitate of MTX-collagen-NPs (MTX-collagen-NPs) was generated by centrifugation at 12,000 rpm for 15 min. The supernatant was immediately harvested after centrifugation to be used in a UV–Vis to measure the absorbance at 377 nm. The procedure was performed three times. An earlier calibration curve was created based on how the absorbance changed with varying MTX concentrations to quantify the free MTX concentration in the solution. Following are the calculations for (DL%) and (EE%) percentages:

6 $$\mathrm{DL }(\mathrm{\%}) = \frac{\mathrm{W }({\text{total}}) -\mathrm{ W}({\text{free}})}{{\text{W}}({\text{collagen}}-{\text{NPs}}) }\times 100\mathrm{\%}$$

7 $$\mathrm{EE }(\mathrm{\%}) = \frac{\mathrm{W }\left({\text{total}}\right)-\mathrm{ W}({\text{free}})}{\mathrm{W }\left({\text{total}}\right) }\times 100\mathrm{\%}$$

where W_(total)_ is the total weight of MTX, W_(free)_ is the total weight of free MTX (not loaded) in the supernatant, and W_(collagen-NPs)_ refers to the total weight of collagen-NPs. The precipitate was washed three times and then lyophilized for evaluation of in vitro drug release.

### In vitro drug release

For the in vitro release behavior evaluation of MTX-collagen-NPs at pH 7.4 (blood pH) and pH 5.5 (acidic intracellular environment), phosphate buffer saline (PBS) was utilized. Dialysis tubing (MWCO; 12–14 kDa) is loaded with 5 mg of MTX-collagen-NPs powder diluted in 5 mL of PBS (pH 7.4 or pH 5.5). The pH was either 7.4 or 5.5, and the dialysis tube was immersed in 40 mL of PBS while being gently stirred at 100 rpm. At predetermined intervals (0, 3, 6, 8, 12, 24, 48 h), an aliquot of 3 mL was removed from the release medium and replaced with an equal aliquot of fresh PBS. The aliquot sample was examined with the UV–Vis spectrometer. The procedure was performed three times. The following equation was applied to determine the cumulative release percentage (CR%) of MTX-collagen-NPs at each time point:

8 $$\mathrm{The\, cumulative\, release\, percentage\, }(\mathrm{CR \%}) = \frac{{\text{Wt}}}{{\text{Wi}}}\times 100$$

where W_t_ is the released drug weight at time _t_ and W_i_ is MTX-collagen-NPs initial weight.

### Ethics approval and consent to participate

All experimental protocols were approved by a Research Ethics Committee, Faculty of Medicine, Mansoura University, Mansoura, Egypt (Code number: MDP.21.01.54). All experiments were carried out in accordance with the applicable laws and regulations. All methods in the study are reported in accordance with ARRIVE guidelines.

## Result and discussion

### Morphological and cultural properties of *Streptomyces* sp. strain NEAA-3

**Figure 2:**
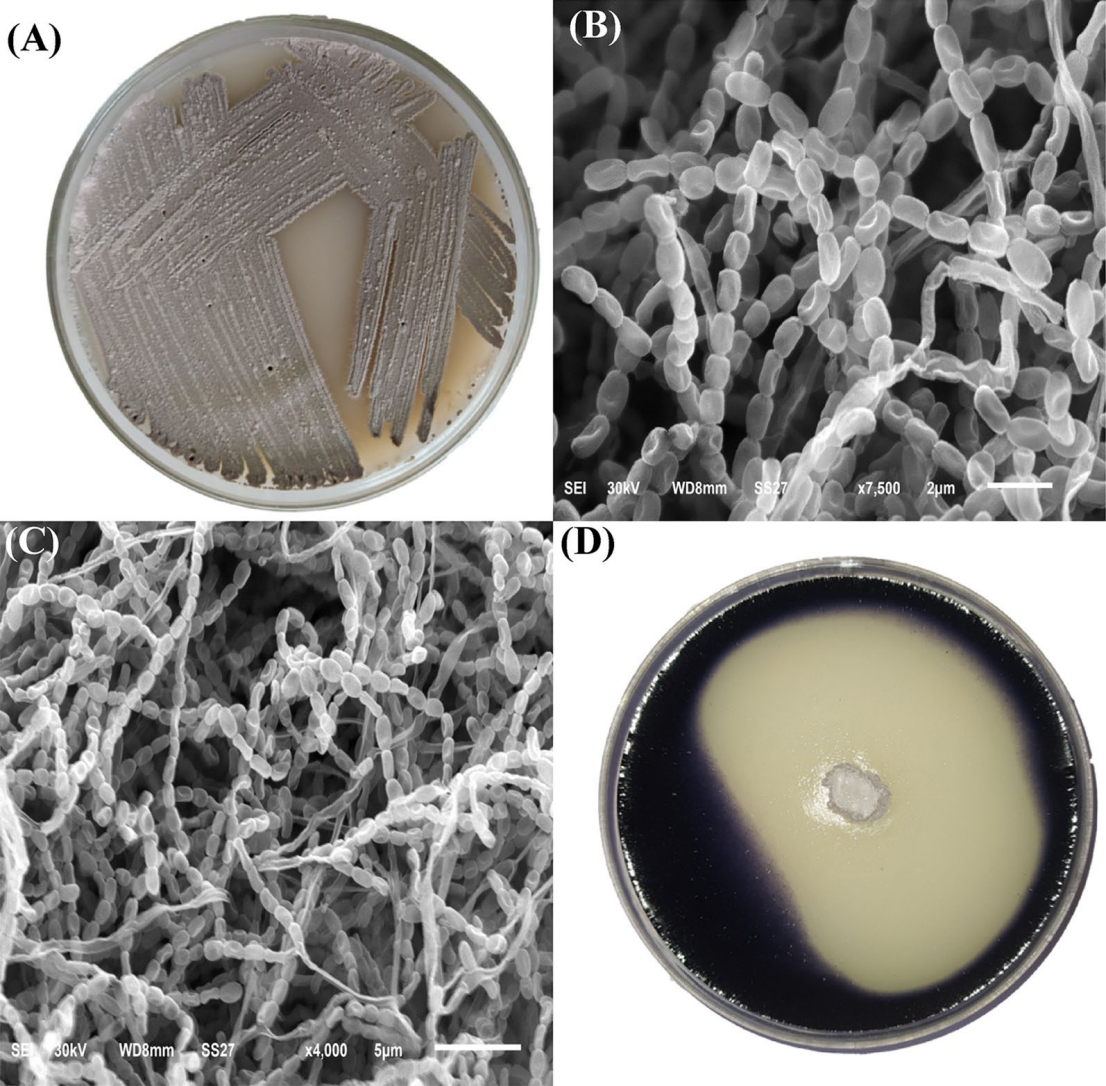
*S. plicatus* NEAA-3 culture on starch nitrate agar (**A**), and scan electron microscopy photos of *Streptomyces plicatus* strain NEAA-3 (**B**,**C**), starch hydrolysis (**D**).

*Streptomyces* sp. strain NEAA-3 is a mesophilic, aerobic, and gram-positive microorganism. *Streptomyces* sp. strain NEAA-3 culture, which had been growing on starch-nitrate agar for 7 days^25^, was examined morphologically (Fig. 2A). Both aerial and substrate mycelium were discovered to be plentiful, well-developed, and not fragmented. Spiral spore chains are carried by aerial mycelium; these spores are non-motile, cylindrical with smooth surfaces (Fig. 2B,C). *Streptomyces* sp. strain NEAA-3 mycelia grew well on the following tested media: tryptone-yeast extract agar, yeast extract-malt extract agar, oatmeal agar, and inorganic salt-starch agar. On the other hand, *Streptomyces* sp. strain NEAA-3 mycelia did not grow on glycerol-asparagine agar and grew poorly on peptone-yeast extract iron agar and tyrosine agar. Faint brown diffusible pigments were observed on starch nitrate agar medium and no diffusible pigments were discovered on the other tested media. On most media, the mature sporulating aerial mycelium ranged in color from gray to brownish gray, but on oatmeal agar, it was reddish gray. The colony's reverse side is a yellowish-brown on all tested media. The substrate pigment is not a pH indicator.

### Physiological properties

The physiological properties of *Streptomyces* sp. strain NEAA-3 are shown in Table 1. No melanin pigments were formed in peptone-yeast-iron agar, glycerol tyrosine agar, or tryptone-yeast agar. It was observed that the starch hydrolysis (Fig. 2D), milk coagulation and gelatin liquefaction recorded positive results. The desired temperature for growth was 30 °C, and the optimal pH level was 7. It was demonstrated that the isolate had a tolerance for NaCl concentrations of up to 7% (w/v). Fructose, ribose, xylose, glucose, maltose, glucose, and lactose were all utilised as the sole carbon source, with the exception of sucrose.

**Table 1:** Phenotypic characteristics that separate *Streptomyces* sp. strain NEAA-3 from other related *Streptomyces* species.

Characteristic	*Streptomyces* sp. NEAA-3	*Streptomyces plicatus*	*Streptomyces enissocaesilis*
Aerial mycelium			
Starch nitrate agar	Brownish gray	Light brownish gray to light grayish reddish brown	Pale yellowish
ISP medium 2	Gray	Light brownish gray to light grayish reddish brown	Light pink-yellowish grayish
ISP medium 3	Reddish gray	Light brownish gray to light grayish reddish brown	
ISP medium 4	Brownish gray	Light brownish gray	light pink-yellowish-grayish
ISP medium 5	None	Light brownish gray	Black grayish brown
ISP medium 6	Light gray	Light brownish gray	
ISP medium 7	Brownish gray	Light brownish gray to light grayish reddish brown	
Reverse side of colony	Yellowish brown	Yellowish brown	
Spore shape	Cylindrical with curved-surfaces	Cylindrical with curved-surfaces	
Spore surface	Smooth	Smooth	Smooth
Spore chain morphology	Spiral	Spiral	Spiral
Melanin production	** − **	−	−
Maximum NaCl conc. (%, w/v) tolerance	7	Up to 10	
Starch hydrolysis	+		
Gelatin liquefaction	+		
Coagulation of milk	+		
Fructose	+++	+	−
Maltose	+++		
Lactose	+++		
Glucose	+	+	+
Sucrose	−	−	−
Ribose	+++		
Xylose	+++	+	+
Galactose	+++		

+ : Positive; − : Negative; Blank cells: no data available.

### Phylogenetic analysis

**Figure 3:**
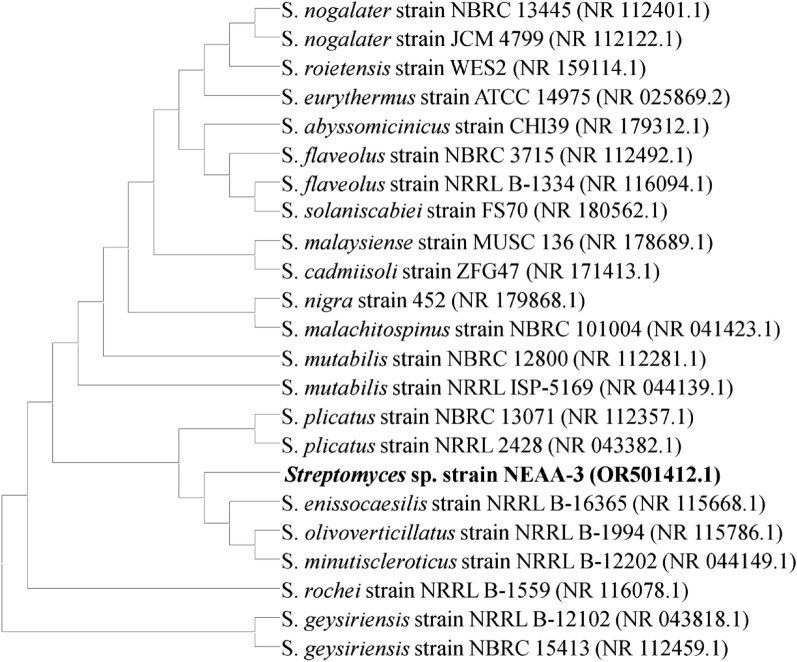
Phylogenetic tree of neighbour-joining constructed on the sequences of 16 S rRNA gene, displaying the relationships between other *Streptomyces* species of related with strain NEAA-3.

The partial 16S rRNA gene sequence for *Streptomyces* sp. strain NEAA-3 (650 bp) was submitted to the GenBank/EMBL/DDBJ databases under the accession number OR501412.1 (https://www.ncbi.nlm.nih.gov/nuccore/OR501412.1?report=GenBank). The 16S rRNA gene sequences of the closest members of the genus *Streptomyces* were compared to those of the *Streptomyces* sp. strain NEAA-3 strain using BLAST search (https://blast.ncbi.nlm.nih.gov/Blast.cgi)^32^. The GenBank database of the BLAST has revealed that several *Streptomyce*s genus species are similar to *Streptomyces* sp. strain NEAA-3. The phylogenetic tree (Fig. 3) was produced using the neighbor-joining technique^33^, which shows that *Streptomyces* sp. strain NEAA-3 is substantially correlated to several other *Streptomyces* species. According to phylogenetic analyses, *Streptomyces* sp. strain NEAA-3 belonged to subclades with *Streptomyces minutiscleroticus* strain NRRL B-12202 (accession No. NR _044149.1) with a similarity of 99.53%; *Streptomyces olivoverticillatus* strain NRRL B-1994 (accession No. NR_115786.1) with a similarity of 99.53%; *Streptomyces plicatus* strain NRRL 2428 (accession No. NR_ 043382.1) with a similarity of 99.69% as well as *Streptomyces enissocaesilis* strain NRRL B-16365 (accession No. NR_115668.1) with a similarity of 99.69%.

It was determined that *Streptomyces* sp. strain NEAA-3 was most closely related to the type strain of *Streptomyces plicatus* strain NRRL 2428 (accession No. NR 043382.1) and *Streptomyces enissocaesilis* strain NRRL B-16365 (accession No. NR_115668.1) with the highest degree of similarity 99.69%^42^, but based on the comparative study based on the previous collected data of the morphological, cultural, and physiological characteristics of the isolate. The isolate was thus named *Streptomyces plicatus* strain NEAA-3. The actinobacteria data for the reference species have been taken from Goodfellow et al.^43^.

### GC–MS spectral analysis of the cell-free supernatant of *Streptomyces plicatus* strain NEAA-3

**Figure 4:**
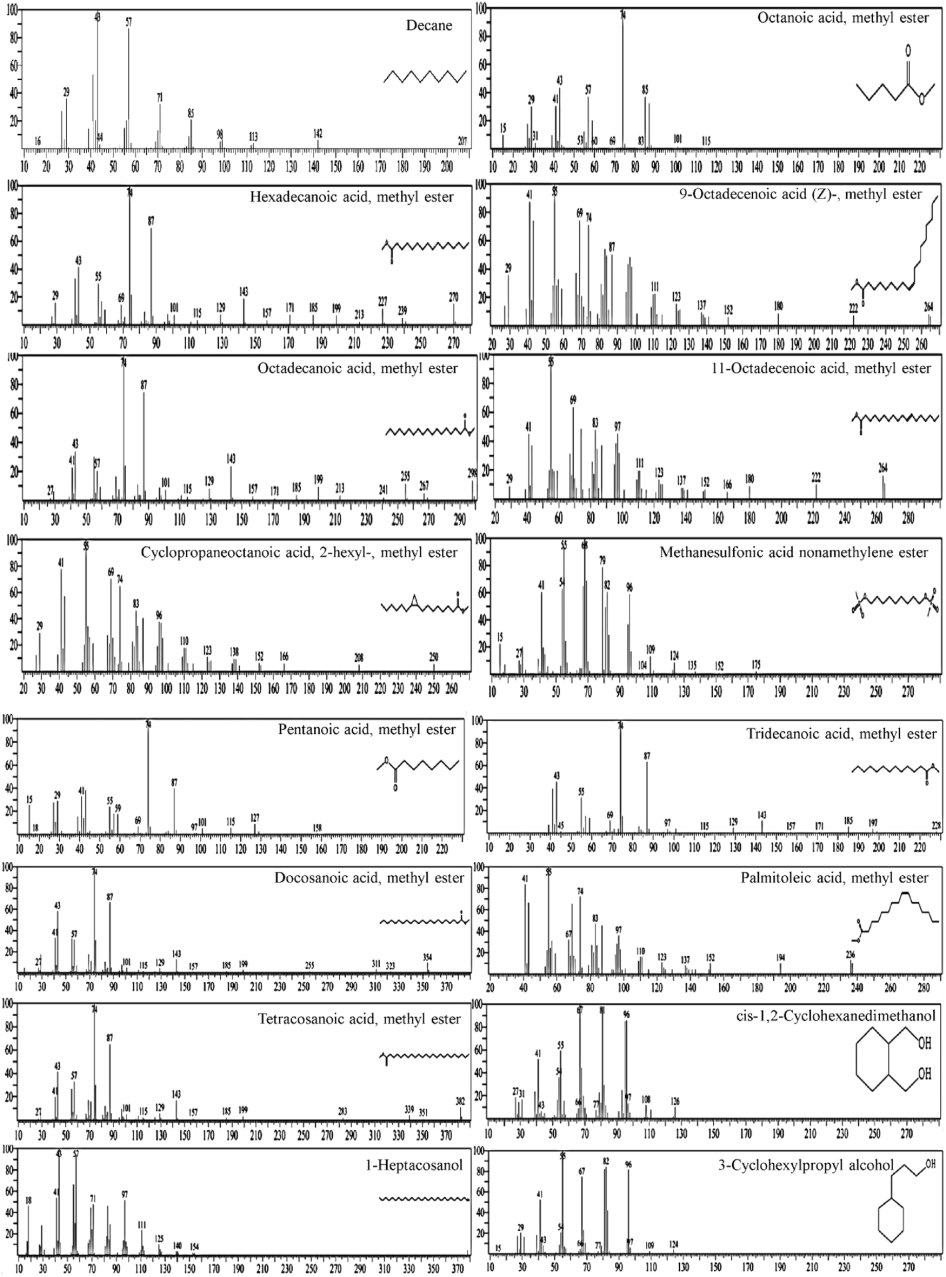
GC–MS chromatogram and chemical composition of bioactive components identified in the methyl acetate of the cell-free supernatant of *Streptomyces plicatus* strain NEAA-3.

GC–MS analysis was performed on the lyophilized cell-free supernatant of *Streptomyces plicatus* strain NEAA-3 that had been dissolved in methanol. A number of fatty acids were identified using GC–MS spectral data (Table 2 and Fig. 4). These fatty acids include linear chain saturated fatty acids methyl ester of; 11-octadecenoic acid, octanoic acid (caprylic acid), pentanoic acid (valeric acid), octadecanoic acid (stearic acid), hexadecanoic acid (palmitic acid), cyclopropaneoctanoic acid, 2-hexyl, ocosanoic acid (behenic acid), tetracosanoic acid (lignoceric acid) and palmitoleic acid. Unsaturated fatty acid; 9-octadecanoic acid (Z) (oleic acid & its isomer elaidic acid) is also present. Methanesulfonic acid nonamethylene also exists. There are three different types of alcohols on display: 1-heptacosanol, cis-1, 2-cyclohexanedimethano and 3-cyclohexylpropyl alcohol. The most prevalent fatty acid was 11-octadecenoic acid, methyl ester, which according to the study of Shoge and Amusan^44^ possesses antibacterial action. Octadecanoic acid, methyl ester (stearic acid) at the third degree was introduced, followed by 9-octadecanoic acid (Z) (oleic acid) methyl ester. Oleic acid, stearic acid and palmitic acid (second degree in abundance) showed antioxidant, in vitro inflammatory and antibacterial activity according to the studies of Fratianni et al.^45^. Caprylic acid is used widely in the food and dairy industries as an antibacterial pesticide for surface sanitization and in the cosmetics, dietary supplements, and other industrial items that promote good health^46^. According to Narang et al.^47^, palmitoleic acid may have the capacity to boost antioxidant enzymes like superoxide dismutase, catalase and glutathione peroxidase. Due to its high acid strength (pKa = − 1.9), methanesulphonic acid is a potent organic acid. Additionally biodegradable and not producing hazardous gases is methanesulfonic acid. Therefore, it was regarded as a green acid^48^.

**Table 2:** Bioactive constituents identified in the cell free supernatant of *Streptomyces plicatus* strain NEAA-3 using GC–MS analysis.

Ret. time (min)	Compound	Peak area (%)	Formula	Mol. wt
8.090	Decane	6.26	C_10_H_22_	142
8.378	Pentanoic acid, methyl ester	1.04	C_6_H_12_O_2_	116
15.120	9-Octadecenoic acid (Z)-, methyl ester	1.71	C_19_H_36_O_2_	296
15.231	Hexadecanoic acid, methyl ester	30.01	C_17_H_34_O_2_	270
16.581	11-Octadecenoic acid, methyl ester	43.12	C_19_H_36_O_2_	296
16.759	Octadecanoic acid, methyl ester	14.73	C_19_H_36_O_2_	296
17.383	Cyclohexanepropanol-	0.61	C_9_H_18_O	142
18.701	9-Octadecenoic acid (Z)-, methyl ester	1.73	C_19_H_36_O_2_	296
18.986	Hexadecanoic acid, 15-methyl-, methyl ester	1.28	C_18_H_36_O_2_	284

### Evaluation of collagen-NPs biosynthesis by the cell-free superntant of *Streptomyces plicatus* strain NEAA-3

**Figure 5:**
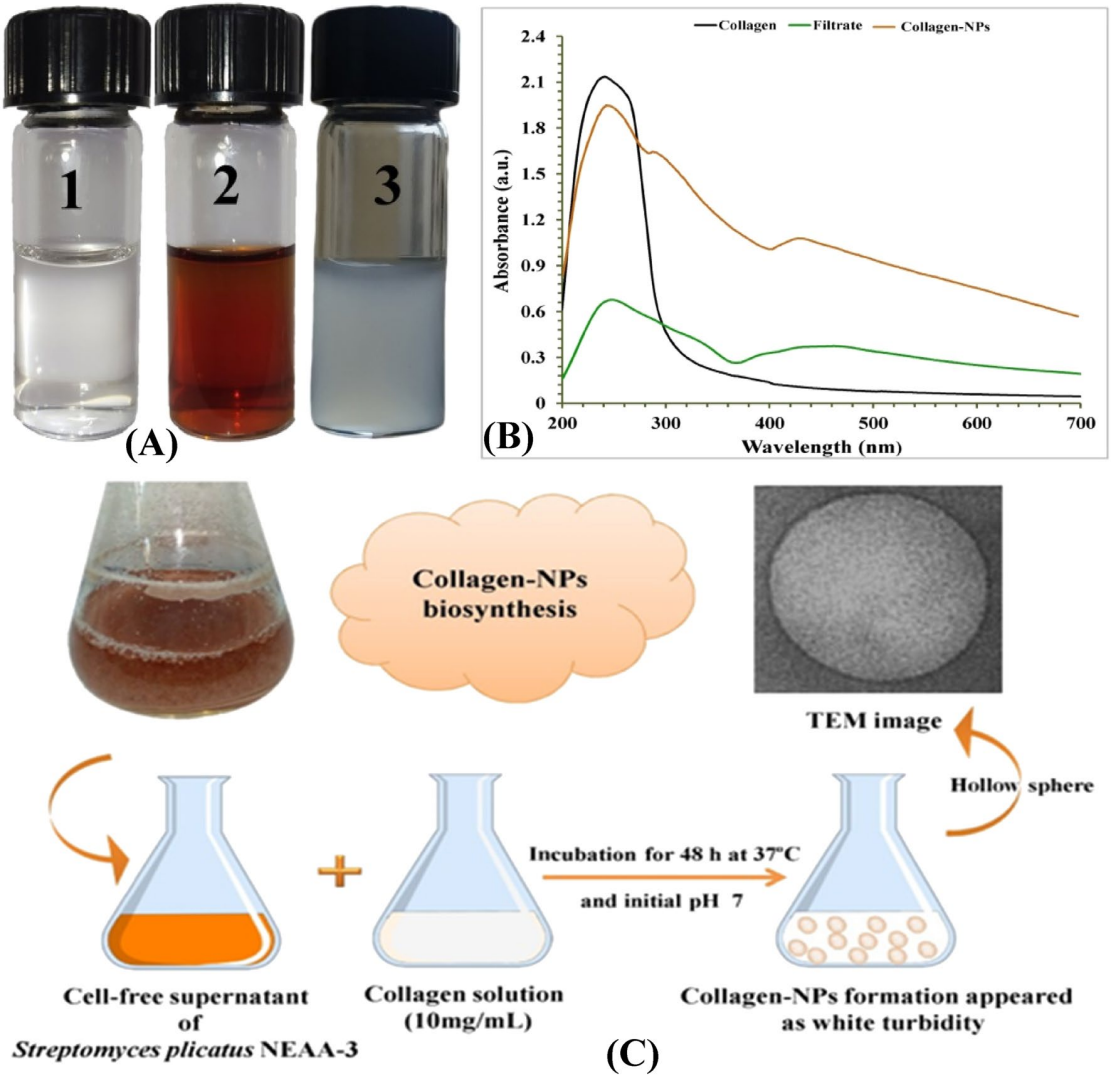
(**A**) Optical observation of collagen soln. (1) Cell-free supernatant of *Streptomyces plicatus* strain NEAA-3 (2), collagen- NPs (3), (**B**) Schematic diagram of collagen-NPs biosynthesis & (**C**) UV–visible absorbance of collagen-NPs biosynthesis by *Streptomyces plicatus* strain NEAA-3.

For the collagen-NPs biosynthesis (Fig. 5A), 1 mL of 10 mg/mL of pure marine collagen solution (type I) was added to 9 mL of freshly prepared cell-free supernatant. Protein nanoparticles can be produced through a process known as desolvation. There are a number of factors that can influence the size of protein nanoparticles during the desolvation process. These factors include the protein concentration, the temperature, the addition of a desolvation agent (like alcohol or natural salt), and the pH of the medium^8^. Consequently, the GC-mass analysis indicates that three different alcohols, including 1-heptacosanol, cis-1, 2-cyclohexanedimethano and 3-cyclohexylpropyl alcohol, can function as a desolvation factor and can be used to bio-fabricate the collagen-NPs. The desolvation factor can alter collagen's structure and reduce its solubility, as seen, for example, in the production of nanoparticles following the addition of glutaraldehyde to collagen mass^49^. Fatty acids and alcohols are thought to change how collagen molecules interact to bio-fabricate the collagen-NPs. Mostly all of the fatty acids found in secondary metabolites have antioxidant characteristics, which means they can reduce collagen protein to bio-fabricate the collagen-NPs. According to the findings of a study conducted by Nagarajan et al.^50^, acetic acid was utilized to bio-fabricate the collagen-NPs. However, methanesulfonic acid nonamethylene is presented in traces; the methanesulfonic acid exhibits a high acidity, which makes it an effective catalyst for chemical processes and collagen-NPs biosynthesis. According to studies conducted by Cinar et al.^51^, Gupta et al.^52^, Xia et al.^53^, and Mourdikoudis et al.^54^, oleic acid is one of the most appealing fatty acids that have been employed in the creation of metal nanoparticles as well as a great capping agent. According to Agrawal et al.^55^, palmitic acid methyl ester was abundant and employed as a superhydrophobic coating material for ZnO nanoparticles.

### Spectroscopy analysis of collagen-NPs

The highest absorption peaks for collagen-NPs, marine collagen solution, and the cell-free supernatant of *Streptomyces plicatus* strain NEAA-3 were determined by UV–visible analyses (Fig. 5B). The highest absorbance peak of marine collagen solution was measured at 240 nm. Saallah et al.^56^ reported that the pure marine collagen type I extracted from *Holothuria scabra* showed two maximum absorption peaks at 240 and 220 nm. Since glycine and proline/hydroxyproline make up the majority of collagen, its greatest absorbance peak falls between 210 and 240 nm^57^, which supports the collagen's purity. Also, the absence of an absorption peak at about 280 nm, which is associated with aromatic amino acids like phenylalanine and tyrosine^58^, further supports collagen's purity. The collagen-NPs' maximal absorption was measured at 240 nm, confirming collagen's presence.

### Microscopy analysis of collagen-NPs

**Figure 6:**
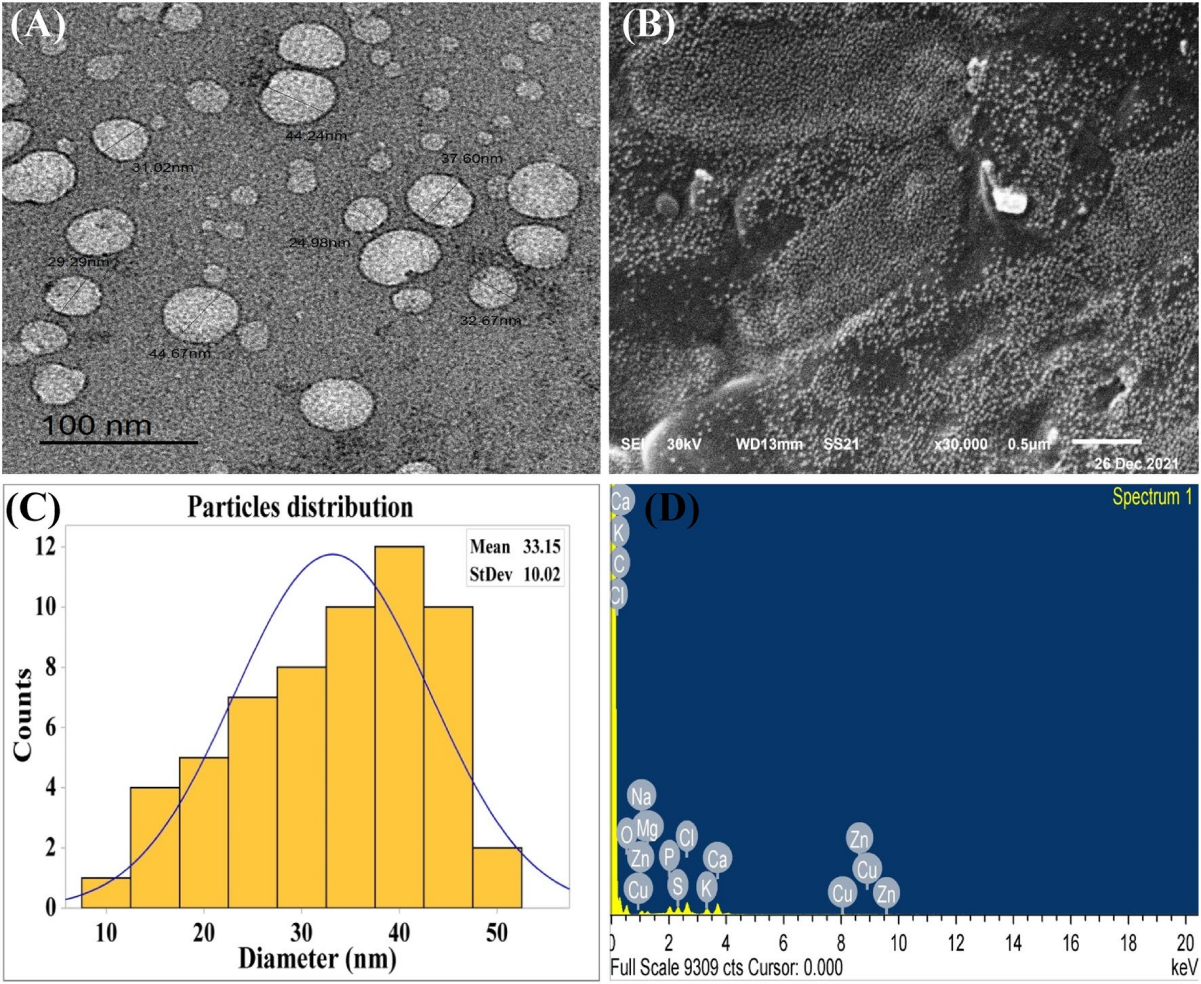
Microscopy investigation of collagen-NPs biosynthesis by *Streptomyces plicatus* strain NEAA-3: (**A**) TEM image, (**B**) SEM image, (**C**) particles size distribution and EDX.

The morphology of collagen-NPs produced using the cell-free supernatant of *Streptomyces plicatus* strain NEAA-3 was investigated and confirmed using TEM and SEM analyses (Fig. 6A,B). The transmission electron micrograph revealed the presence of hollow sphere nanoparticles (Fig. 6C). The mean diameter of the produced collagen-NPs was recorded as 33.15 ± 10.02 nm, which is less than 100. The small size of nanoparticles is advantageous, especially in the medical applications. The small sizes of nanoparticles allow them to not only transport widely throughout the body, but they can also penetrate cells or be modified to attach to specific types of cells. Collagen-NPs can be an ideal 3D biomaterial due to their nanoscale size between 1 and 100 nm. It also has a high surface area-to-volume ratio that facilitates effective interaction and penetration into wound sites^59^.

In the EDX results of the collagen-NPs powder (Fig. 6D), the elemental peaks for C, O_2_, Na, Mn, Si, P, S, Cl, K, Ca, Cu, and Zn were observed. Carbon was the most abundant element, with weight and atomic percentages of 58.83 and 68.81, respectively, followed by oxygen with values of 29.96 and 26.31, respectively.

### Fourier-transform infra-red (FTIR)

**Figure 7:**
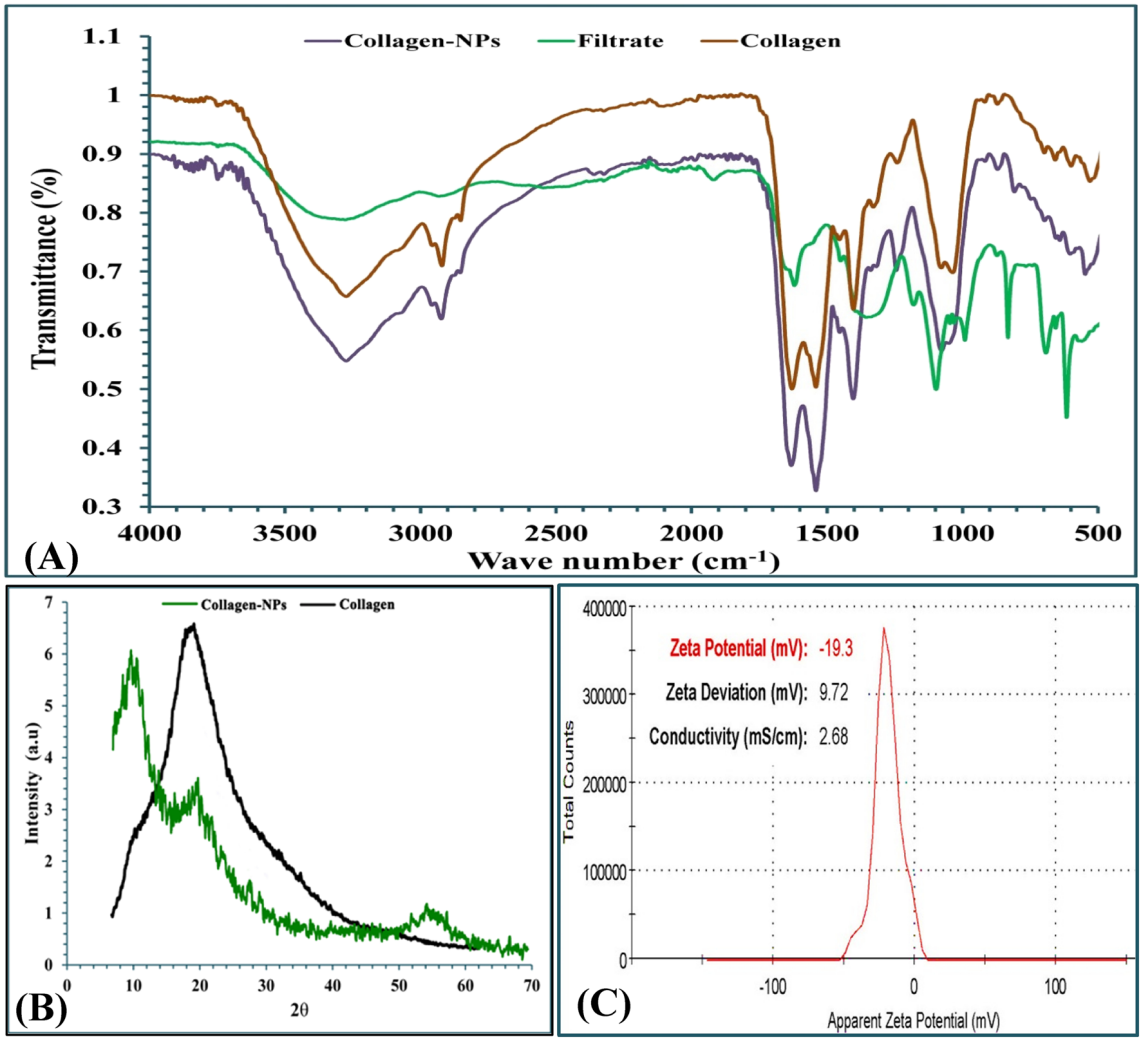
FTIR (**A**), X-ray diffraction analysis (**B**) and Zeta-potential (**C**) of collagen-NPs by biosynthesis by *Streptomyces plicatus* strain NEAA-3.

For many years, researchers have successfully used FTIR analysis (Fig. 7A) to examine the structural dynamics, conformational changes, secondary structures, and stability studies of proteins^60^. The produced collagen-NPs revealed several absorption peaks in the FTIR spectrum (3304, 2934, 1641, 1546, 1409, 1245, 1081, 933 and 619 cm^−1^). The peak at 3304 cm^−1^ belongs to the vibrations of N–H stretching that refer to NH_2_ in aromatic amines, amide A and primary amines that present between 3500 cm^−1^ and 3300 cm^−1^ according to Pati et al.^61^ research. Amide B refers to CH of symmetric and anti-symmetric stretching that is present in CH_3_ and CH_2_ in aliphatic compounds and is detected in the absorption peak at 2934 cm^−1^, which can be observed in the range of 2990 to 2850 cm^−1^ according to Movasaghi et al.^62^. The amide I bond coupled with the (C=O) group straight with the polypeptide backbone was the distinctive frequency marker of peptide secondary structure found between 1600 and 1700 cm^−1^ according to Haris & Severcan^63^, which was identified at 1641 cm^−1^. The peak at 1546 cm^−1^, which is found between the amide II's typical absorption ranges (1478–1565 cm^−1^), belongs to NH in secondary amides^64^. The C–C stretch of aromatic compounds is the source of the 1409 cm^−1^ peak. The absorption peak at 1245 cm^−1^ was discovered to fall within the 1411 cm^−1^ (amide II) and 1241 cm^−1^ (amide III) band ratio range, which was almost equal to 1, confirming the collagen triple helical structure^65^. The peak at 1081 cm^−1^ demonstrated that the C–O stretch in alcohol exists. Peaks at 933 cm^−1^, and 619 cm^−1^ all provided evidence for the occurrence of 1,2,4-trisubust benzene, Ar–OH in phenols, and O–C=O in carboxylic acid, respectively^64^.

### Analysis of X-ray Diffraction (XRD)

The XRD diagram is displayed in Fig. 7B. At diffraction angles (2*θ*) of 10° and 24°, collagen-NPs exhibited two typical peaks that matched the characteristics diffraction peaks of pure collagen^66^. The first peak is related with the triple helical structure of collagen, whereas the second peak is related with the distance between skeletons. Diffraction peaks can be used to determine the ordered structures of collagen^67^.

### The Zeta potential

Zeta potential can be negative or positive and plays a key role in determining the stability of the nanoparticles in colloidal solutions.The surface charges on the particles may influence the stability of the nanoparticle’s preparation through substantial electrostatic repulsion between the particles^68^. Marine collagen type I nanoparticles have a limited range of Zeta potential, which increases surface area, dispersion capacity, and catalytic activity^69^. The Zeta potential value was recorded at − 19.3 mV, with a Zeta deviation of 9.72 mV and conductivity of 2.68 mS/cm, as shown in Fig. 7C. Zeta potential value of the biosynthesized collagen-NPs produced by the cell-free supernatant of *Streptomyces xinghaiensis* NEAA-1 was − 18.4 mV, which coordinates with our result^70^. The results of this study indicated that the biosynthesized collagen-NPs using the cell-free supernatant of *Streptomyces plicatus* strain NEAA-3 had notably superior stability to that of the chemically synthesized collagen-NPs.

### Thermogravimetric analysis (TGA)

**Figure 8:**
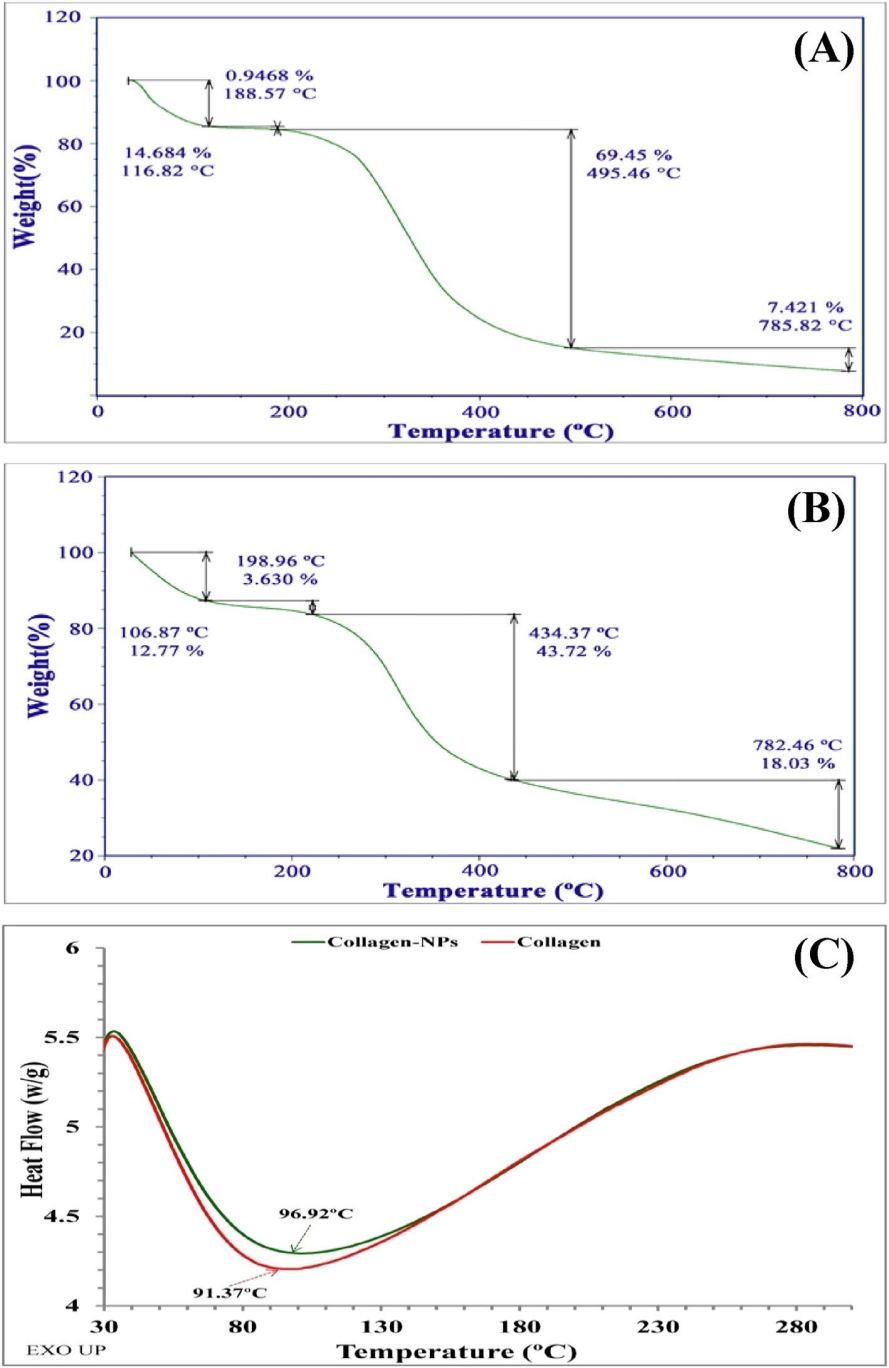
Thermal stability investigation of collagen-NPs: TGA analysis for collagen-NPs (**A**) and collagen (**B**). DSC of collagen-NPs and collagen.

The thermal stability of polymeric nanoparticles refers to their ability to resist heat and maintain their properties, such as strength, toughness, or elasticity, at a particular temperature^71^. TGA is typically used to test the thermal stability of collagen-NPs. Figure 8A displays the TGA analysis. The first transition phase of collagen-NPs was initiated by the first weight loss of the particles at a temperature of 116.82 °C and a weight percentage of 14.684. The weight percentage decreased to 12.77 and the transition temperature shifted to 106.87 °C when compared to collagen after thermal degradation (Fig. 8B). According to a study by Ebnesajjad et al.^72^, the first weight loss is attributed to the samples' moisture content and volatile components. The second transition temperature of collagen occurred at 198.96 °C with a weight percentage of 3.63, while the second transition temperature of collagen-NPs occurred at 188.57 °C with a weight percentage of 0.9468. The second decomposition phase is caused by the decomposition of collagen and the consumption of collagen organic matrix waste^73^.

TGA curves for pure collagen and mineralized collagen revealed the same profile, with weight loss due to the evaporation of physisorbed water between room temperature and 200 °C, and weight loss related to the decomposition of collagen molecules between 200 and 500 °C and the combustion of the remaining organic components causing a minor loss of weight between 500 and 700 °C^74,75^. The third transition phase is regarded to be the maximum denaturation temperature for both collagen and collagen-NPs. The remaining weight percentages of collagen and collagen-NPs were 43.72 and 69.45, respectively, and their corresponding transition temperatures were 434.37 and 495.46, respectively. The findings of this research demonstrated that the collagen-NPs exhibited enhanced thermal stability due to the pervasive presence of bioactive components on their surfaces as capping agents. The remaining final weight percentages of collagen and collagen-NPs were 18.03% and 7.421%, respectively and their corresponding transition temperatures were 782.46 °C and 785.82 °C, respectively due to the combustion of the remaining organic components according to Tampieri et al*.*^74^.

### Differential scanning calorimetry (DSC)

DSC approach is a direct method that can reveal details about the actual thermodynamic properties of protein thermal transitions and the evaluation of variables that are crucial to the stability of certain proteins^76^. Theoretically, information about the thermal transitions of proteins can be obtained by measuring the heat flow between the sample (collagen-NPs) and reference (collagen). At the greatest transition point (Tmax), also known as the endothermic peak of transition curves, the transition temperature value for both collagen-NPs and the collagen sample was calculated. Figure 8C demonstrated an endothermic peak at 96.92 °C, which was greater than 91.37 °C for collagen; this is related to the disintegration of the triple helical structure as well as the release of the water-bound collagen molecule^77^. The high endothermic temperature peak of collagen-NPs is assumed to be caused by the bioactive compounds present in the capping agents, which were derived from the cell-free supernatant of *Streptomyces plicatus* strain NEAA-3.

### Face-centered central composite for optimizing the biosynthesis of collagen-NPs

The biosynthesis of collagen-NPs was optimized using a face-centered central composite. Table 3 lists the results of 30 trials that used with different combinations of the following four variables: collagen concentration, pH, temperature and incubation time (X_1_, X_2_, X_3_ and X_4_; respectively). Run orders 4, 11, 17, 26, and 30 showed the highest production of collagen-NPs. The maximal collagen-NPs biosynthesis occurred at a value of 9.33 mg/mL, which appeared in run order 30 under the conditions of 10 mg/L of collagen concentration, 72 h of incubation, initial pH of 7, and 35 °C. The lowest biosynthesis of collagen-NPs was noticed in run order 21, which achieved 1.8 mg/mL, under the condition of 5 mg/mL of collagen concentration, initial pH of 5, 24 h of incubation time and 25 °C. Experimented and predicted values are presented in Table 3.

**Table 3:** Face-centered central composite design matrix representing collagen-NPs biosynthesis by *Streptomyces plicatus* strain NEAA-3 as affected by pH, temperature, collagen concentrations and incubation time with factor levels (coded and actual).

Std	Run	Variables				Collagen-NPs biosynthesis (mg/mL)		Residuals
		X_1_	X_2_	X_3_	X_4_	Actual	Predicted	
3	1	− 1	1	− 1	− 1	3.69	3.61	0.08
22	2	0	0	1	0	7.33	7.47	− 0.14
6	3	1	− 1	1	− 1	7.37	7.35	0.02
29	4	0	0	0	0	9.29	9.07	0.22
9	5	− 1	− 1	− 1	1	4.05	3.99	0.06
18	6	1	0	0	0	8.80	8.94	− 0.14
14	7	1	− 1	1	1	6.92	6.95	− 0.03
4	8	1	1	− 1	− 1	6.43	6.35	0.08
7	9	− 1	1	1	− 1	2.81	2.76	0.05
8	10	1	1	1	− 1	6.92	6.93	− 0.01
25	11	0	0	0	0	9.21	9.07	0.14
15	12	− 1	1	1	1	2.33	2.37	− 0.04
20	13	0	1	0	0	7.72	7.90	− 0.18
24	14	0	0	0	1	8.74	8.65	0.09
11	15	− 1	1	− 1	1	3.11	3.12	− 0.01
17	16	− 1	0	0	0	5.20	5.34	− 0.14
30	17	0	0	0	0	9.28	9.07	0.21
23	18	0	0	0	− 1	8.72	9.09	− 0.37
16	19	1	1	1	1	5.44	5.35	0.09
13	20	− 1	− 1	1	1	2.42	2.48	− 0.06
5	21	− 1	− 1	1	− 1	1.80	1.68	0.12
21	22	0	0	− 1	0	7.80	7.94	− 0.14
12	23	1	1	− 1	1	4.59	4.66	− 0.07
10	24	1	− 1	− 1	1	7.00	7.03	− 0.03
2	25	1	− 1	− 1	− 1	7.62	7.53	0.09
28	26	0	0	0	0	9.05	9.07	− 0.02
26	27	0	0	0	0	9.11	9.07	0.04
1	28	− 1	− 1	− 1	− 1	3.22	3.29	− 0.07
19	29	0	− 1	0	0	8.44	8.54	− 0.10
27	30	0	0	0	0	9.33	9.07	0.26

Variable (g/L)	Code	Coded and actual levels						
		− 1	0	1				
Collagen conc. (mg/mL)	X_1_	5	10	15				
pH	X_2_	5	7	9				
Temperature (°C)	X_3_	25	35	45				
Incubation time (h)	X_4_	24	72	120				

### ANOVA and multiple regression analysis

Table 4 displays the ANOVA results for the experimental design, the Fisher (*F*-value), the probability (*P*-value). Given that the *F*-value of the model is 379.36, with a very low *P*-value (< 0.0001) it can be concluded that the model is statistically very significant (Table 4). The model terms are significant when the *P*-value is less than 0.05. Coefficients with smaller *P-*values are more significant^78^. With a *P*-value of less than 0.0001, almost all parameters of the linear effect (X_1_, X_2_, X_3_, X_4_), interaction effect (X_1_X_2_, X_1_X_3_, X_1_X_4_, X_2_X_3_, X_2_X_4_) and quadric effect (X_1_^2^, X_2_^2^, X_3_^2^) demonstrated maximum significance suggesting that the model terms are significant. While X_3_X_4_ (linear effect) and X_4_^2^ (quadric effect) are not significant.

**Table 4:** Variance analysis for collagen-NPs biosynthesis by *Streptomyces plicatus* strain NEAA-3 as affected by pH, incubation time, collagen concentrations and temperature with factor levels (coded and actual).

Source of variance		Coefficient estimate	Sum of squares	Degrees of freedom	Mean square	*F*-value	*P*-value
Model		9.07	181.45	14	12.96	379.36	< 0.0001
Linear effect	X_1_	1.80	58.54	1	58.54	1713.32	< 0.0001
	X_2_	− 0.32	1.87	1	1.87	54.70	< 0.0001
	X_3_	− 0.23	0.97	1	0.97	28.28	< 0.0001
	X_4_	− 0.22	0.88	1	0.88	25.76	0.0001
Interaction effect	X_1_X_2_	− 0.37	2.24	1	2.24	65.42	< 0.0001
	X_1_X_3_	0.36	2.04	1	2.04	59.85	< 0.0001
	X_1_X_4_	− 0.30	1.43	1	1.43	41.80	< 0.0001
	X_2_X_3_	0.19	0.59	1	0.59	17.13	0.0009
	X_2_X_4_	− 0.30	1.42	1	1.42	41.45	< 0.0001
	X_3_X_4_	0.03	0.01	1	0.01	0.32	0.5784
Quadratic effect	X_1_^2^	− 1.93	9.68	1	9.68	283.38	< 0.0001
	X_2_^2^	− 0.85	1.89	1	1.89	55.19	< 0.0001
	X_3_^2^	− 1.37	4.85	1	4.85	141.93	< 0.0001
	X_4_^2^	− 0.20	0.11	1	0.11	3.13	0.0973
Error effect	Lack of Fit		0.45	10	0.05	3.68	0.0816
	Pure Error		0.06	5	0.01		
R^2^	0.9972	Std. Dev	0.18				
Adj R^2^	0.9946	Mean	6.46				
Pred R^2^	0.99	C.V. %	2.86				
Adeq Precision	56.71						

*C.V: Coefficient of variation, *P*: Level of significance, *F*: Fishers's function.

According to the study of El-Naggar et al.^20^, there is a perfect correlation between predicted and actual response values when the R^2^-value is more than 0.9. Consequently, the R^2^-value of the model (0.9972) demonstrated a robust correlation between the observed and predicted values, which suggests that the current model is reliable for the biosynthesis of collagen-NPs. The adjusted R^2^ of 0.9946 and the predicted R^2^ of 0.99 are reasonably in agreement; hence, the difference is less than 0.2^79^.

The coefficient estimations also revealed whether collagen-NPs were changed positively or negatively. Antagonism (negative coefficient) and synergism (positive coefficient) are the two possible interactions between two variables^80^. A large estimated coefficient, whether positive or negative, indicates that the independent factors significantly affect the response. Production increases at high levels of every investigated variable whose expected coefficient has a positive sign^81^. If the sign is negative, it is implied that production increases when the variable is present at low levels. Table 4 shows the positive coefficient parameters (X_1_ as a linear effect and X_1_X_3_, X_2_X_3_ and X_3_X_4_ as an interaction effect) that increased the production of collagen-NPs. While all other coefficients are negative, the positive coefficient for X_1_ indicates that this variable increased collagen-NPs production linearly.

The statistical analysis of collagen-NPs biosynthesis reveals a 2.86% coefficient of variation percentage (C.V. %), which is comparatively very low and indicates great accuracy, dependability, and precision of experimental trials. A relatively small value of the coefficient of variation % reflects high precision and accuracy of the experimental values^82^. The ratio of signal to noise is measured by Adeq Precision. A ratio higher than 4 is desirable since it indicates that the model has high accuracy^83^. The model's precision is 56.71 in the present investigation, indicating the model's precision. The mean of the model is 6.46, with a standard deviation of 0.18.

The fit summary results provided in Table 5 were utilized to choose between the 2FI, linear and quadratic models as the most suitable polynomial model for collagen-NPs biosynthesis by the cell-free supernatant of *Streptomyces plicatus* strain NEAA-3. Since the lack of fit is not statistically significant (*P*-value is 0.0816 and the *F*-value is 3.68), the quadratic model is an appropriate model for the collagen-NPs biosynthesis by the cell-free supernatant of *Streptomyces plicatus* strain NEAA-3. Furthermore, it is observed that the quadratic model has R^2^ value of 0.9972, adjusted R^2^ value of 0.9946 and predicted R^2^ value of 0.99, all of which are higher than the 2FI and linear models (Table 5). The quadratic model has a lower standard deviation of 0.18 and PRESS value of 1.82.

**Table 5:** Fit summary for face-centered central composite design results for collagen-NPs biosynthesis by *Streptomyces plicatus* strain NEAA-3 as affected by pH, incubation time, collagen concentrations and temperature with factor levels (coded and actual).

Source	Sum of Squares	*Df*	Mean Square	*F-*value	*P-*value *P*rob > *F*
Lack of fit tests					
Linear	119.65	20	5.98	488.11	< 0.0001
2FI	111.93	14	8.00	652.31	< 0.0001
Quadratic	0.45	10	0.05	3.68	0.0816
Sequential model sum of squares					
Linear vs Mean	62.25	4	15.56	3.25	0.0281
2FI vs Linear	7.72	6	1.29	0.22	0.9662
Quadratic vs 2FI	111.48	4	27.87	815.75	< 0.0001

Model summary statistics					
Source	Standard deviation	R-Squared	Adjusted R-Squared	Predicted R-Squared	PRESS
Linear	2.19	0.3421	0.2368	0.0585	171.33
2FI	2.43	0.3845	0.0606	-1.3126	420.82
Quadratic	0.18	0.9972	0.9946	0.99	1.82

* Significant values, *df*: degree of freedom, PRESS: sum of squares of prediction error, 2FI: two factors interaction.

The maximum collagen-NPs biosynthesis by the cell-free supernatant of *Streptomyces plicatus* strain NEAA-3 that correspond to the four variables' optimum levels were determined using the second-order polynomial model (Eq. 9), and the relationship between collagen-NPs biosynthesis and the independent variables (pH, temperature, incubation time, and collagen concentration) was assessed. The predicted collagen-NPs biosynthesis (Y) that correspond to the four independent variables was calculated as follows.

9 $${\text{Y}} = { 9}.0{7} + { 1}.{\text{8X}}_{{1}} - \, 0.{\text{32X}}_{{2}} - \, 0.{\text{23X}}_{{3}} - \, 0.{\text{22X}}_{{4}} - \, 0.{\text{37X}}_{{1}} {\text{X}}_{{2}} + \, 0.{\text{36X}}_{{1}} {\text{X}}_{{3}} - \, 0.{\text{3X}}_{{1}} {\text{X}}_{{4}} + \, 0.{\text{19X}}_{{2}} {\text{X}}_{{3}} - \, 0.{\text{3 X}}_{{2}} {\text{X}}_{{4}} + \, 0.0{\text{3X}}_{{3}} {\text{X}}_{{4}} - { 1}.{\text{93 X}}_{{1}}^{{2}} - \, 0.{\text{85X}}_{{2}}^{{2}} - {1}.{\text{37X}}_{{3}}^{{2}} - 0.{\text{2 X}}_{{4}}^{{2}}$$

### The model adequacy

**Figure 9:**
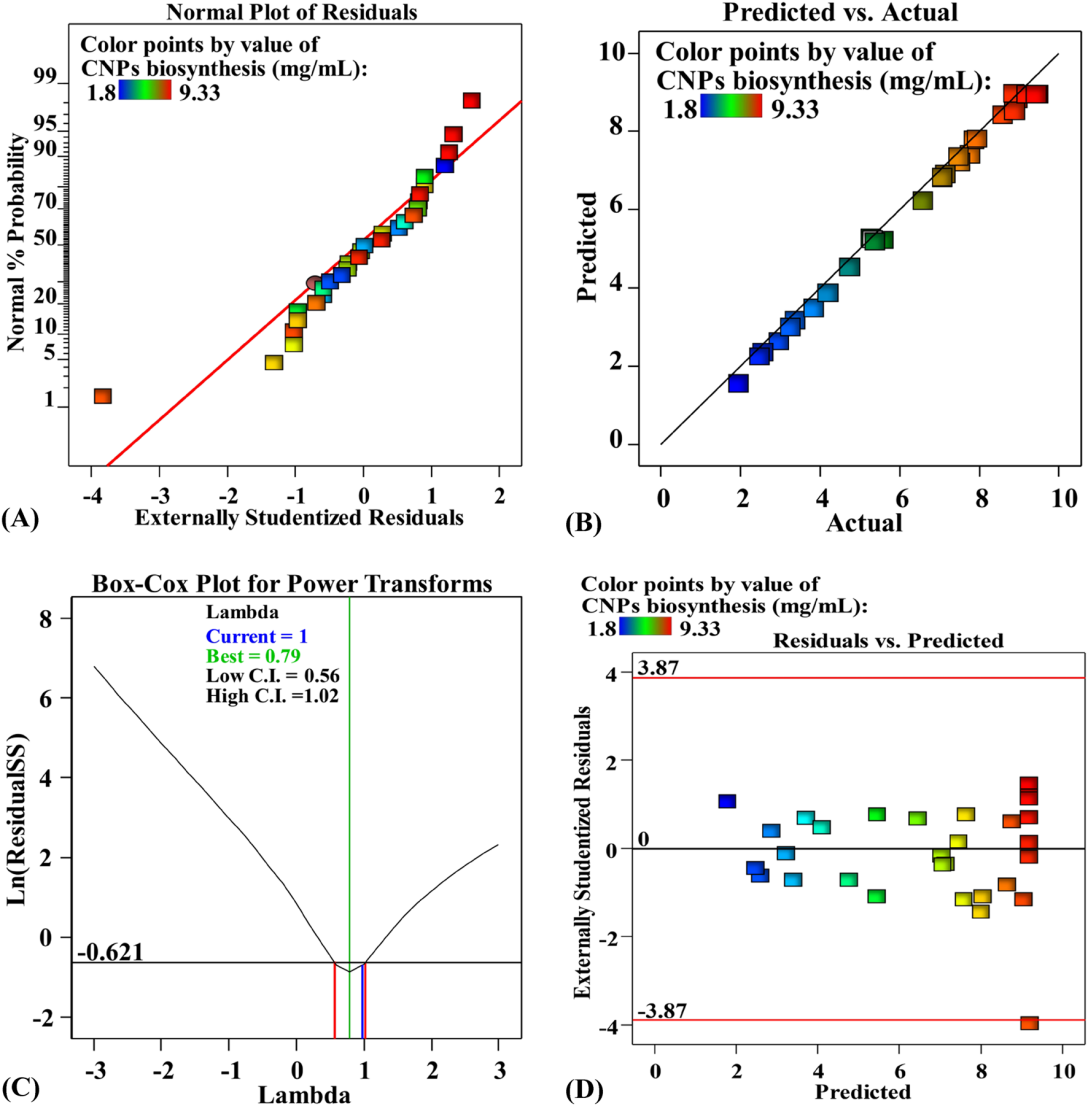
(**A**) Normal probability plotting of internally studentized residuals, (**B**) plotting of actual versus predicted (**C**) Box-Cox plotting of model transformation and (**D**) plotting of internally studentized residuals versus predicted values of collagen-NPs biosynthesis by *Streptomyces plicatus* strain NEAA-3. CNPs: collagen-NPs.

The normal probability plot (NPP) is a statistic tool that indicates whether or not a model's residuals follow a normal distribution^36^. The difference between theoretical and experimental data is referred to as the residuals^84^, and a small residual indicates a high degree of model correctness. NPP is displayed in Fig. 9A, indicating that the residuals are found on the diagonal straight line of collagen-NPs biosynthesis by the cell-free supernatant of *Streptomyces plicatus* strain NEAA-3. This demonstrates that the predicted data fit the experimental findings, confirming the model's accuracy^19,85^.

Figure 9B demonstrated the relationship between the predicted and experimental collagen-NPs biosynthesis values. It demonstrates that all point is located in close proximity to the prediction line, indicating that the model is adequate and explains the excellent agreement between the experimental and theoretical results^86,87^.

The Box-Cox graph of model transformation graph is displayed in Fig. 9C. The red and blue lines show the minimum and maximum confidence intervals (C.I.) values (0.56–1.02; respectively), and the blue line shows the current lambda value (lambda = 1). The green line represents the best lambda value (best lambda = 0.79). The blue line lies in the optimal zone between the minimum and maximum C.I., this indicates that the predicted data fits the experimental data well and confirming that no data transformation is required^19,88^.

The externally studentized residuals were plotted against the predicted values of collagen-NPs biosynthesis by the cell-free supernatant of *Streptomyces plicatus* strain NEAA-3 in Fig. 9D. It shows the residuals against the predicted values. The residual points are scattered randomly nearly along the zero line, which indicates that the values of the experimental results have a constant small variation from the predicted data^89,90^.

### Three-dimensional surface plots

**Figure 10:**
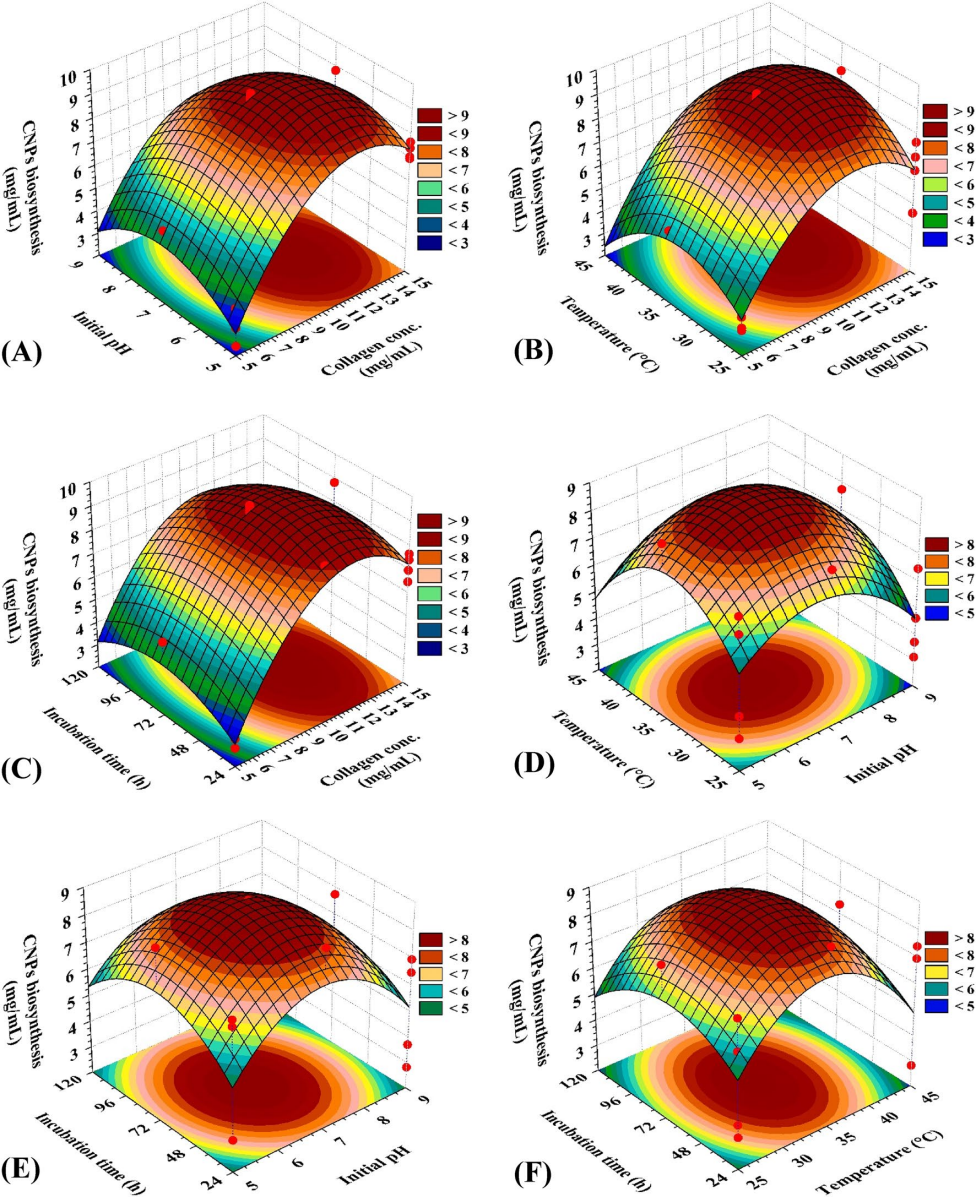
3D plots showing the mutual effects of collagen concentration (X_1_), pH (X_2_), temperature (X_3_) and incubation time (X_4_). on collagen nanoparticles biosynthesis by *Streptomyces plicatus* strain NEAA-3. CNPs: collagen-NPs.

The four independent bioprocess variables were combined in pairwise combinations to assess their optimal levels and their mutual interaction effects on collagen-NPs biosynthesis by the cell-free supernatant of *Streptomyces plicatus* strain NEAA-3. These three-dimensional graphs are shown in Fig. 10A–F. Two of the independent variables were plotted against collagen-NPs biosynthesis on the Z-axis while the other two independent variables held at their zero levels. The in vitro synthesis of collagen nanostructures is reported to be influenced by temperature, pH, ionic strength, collagen type or concentration, and pH^90^.

Figure 10A displays the relationship between collagen-NPs biosynthesis and the mutual interactions between X_1_ (collagen concentration) and X_2_ (initial pH), whereas X_3_ (temperature) and X_4_ (incubation time) had been maintained at zero levels (35 °C and 72 h; respectively). Collagen-NPs biosynthesis value increased gradually until the optimal pH (7) was reached, and decreasing or increasing the pH value, the collagen-NPs biosynthesis will be decreased. By increasing collagen concentration, the collagen-NPs biosynthesis increased up to 10–12 mg/mL. However, the biosynthesis of collagen-NPs is gradually reduced by subsequent increases in collagen concentration. Collagen nanostructures were produced in numerous studies using pH values between 7 and 7.4. Collagen type I has a pH of 7.2, which is close to the isoelectric point where the charge of the collagen amino acids is balanced, favoring the creation of nanostructures^91^.

Figure 10B displays the relationship between collagen-NPs biosynthesis and the mutual interactions between X_1_ (collagen concentration) and X_3_ (temperature), whereas X_2_ (pH) and X_4_ (incubation time) had been maintained at zero levels (7 pH and 72 h; respectively). Collagen-NPs increase gradually as the temperature and collagen concentration increase until they reach their midpoints (10 mg/mL and 35 °C; respectively). However, the biosynthesis of collagen-NPs is gradually reduced by subsequent increases in temperature or collagen concentration due to the high concentration of collagen and the supernatant's incapacity to transform the collagen protein to collagen-NPs. Room temperature, which was between 35 and 37 °C, was the ideal temperature for the synthesis of collagen-NPs. According to Luo et al.^11^, the collagen-like peptide was able to create well-defined nano-vesicles with diameters of about 100 nm and a transition temperature of 37 °C.

Figure 10C displays the relationship between collagen-NPs biosynthesis and the mutual interactions between X_1_ (collagen concentration) and X_4_ (incubation time), whereas X_2_ (initial pH) and X_3_ (temperature) had been maintained at zero levels (7 pH and 35 °C; respectively). The collagen-NPs biosynthesis increased rapidly as the concentration of collagen and the incubation time increased. This continued until their midpoints (10 mg/mL and 72 h; respectively) were reached. After that point, collagen-NPs biosynthesis decreased due to the high concentration of collagen and the incapacity of the supernatant transform the collagen to collagen-NPs. In addition, the prolonged incubation period caused agglomeration of nanoparticles, which had a negative impact on collagen-NPs biosynthesis.

Figure 10D displays the relationship between collagen-NPs biosynthesis and the mutual interactions between X_2_ (initial pH) and X_3_ (temperature), whereas X_1_ (collagen concentration) and X_4_ (incubation time) had been maintained at zero levels (10 mg/mL and 72 h; respectively). The collagen-NPs increased as the temperature and initial pH level were increased until they reached their midpoints. Collagen-NPs will then decrease as initial pH and temperature increase. According to Song et al.^92^, too much acid or alkali can cause collagen molecules to lose their ionic and hydrogen bonds. The pH of the media is the main determining factor since it can affect the charge distribution and electrostatic interactions of the collagen molecules, affecting their ability to form nanostructures^93^.

Figure 10E displays the relationship between collagen-NPs biosynthesis and the mutual interactions between X_2_ (initial pH) and X_4_ (incubation time), whereas X_1_ (collagen concentration) and X_3_ (temperature) had been maintained at zero levels (10 mg/mL and 37 °C; respectively). As the initial pH and incubation time increased, collagen-NPs production gradually increased until it reached the midpoint. The collagen-NPs aggregated as a result of the prolonged incubation time.

Figure 10F displays the relationship between collagen-NPs biosynthesis and the mutual interactions between X_3_ (temperature) and X_4_ (incubation time), whereas the X_1_ (collagen concentration) and X_2_ (initial pH) had been maintained at zero levels (10 mg/mL and 7 pH; respectively). The collagen-NPs increase significantly as the temperature and incubation time increase until they reached their midpoints. Collagen-NPs will then decrease as incubation time and temperature increase.

### Desirability function analysis

**Figure 11:**
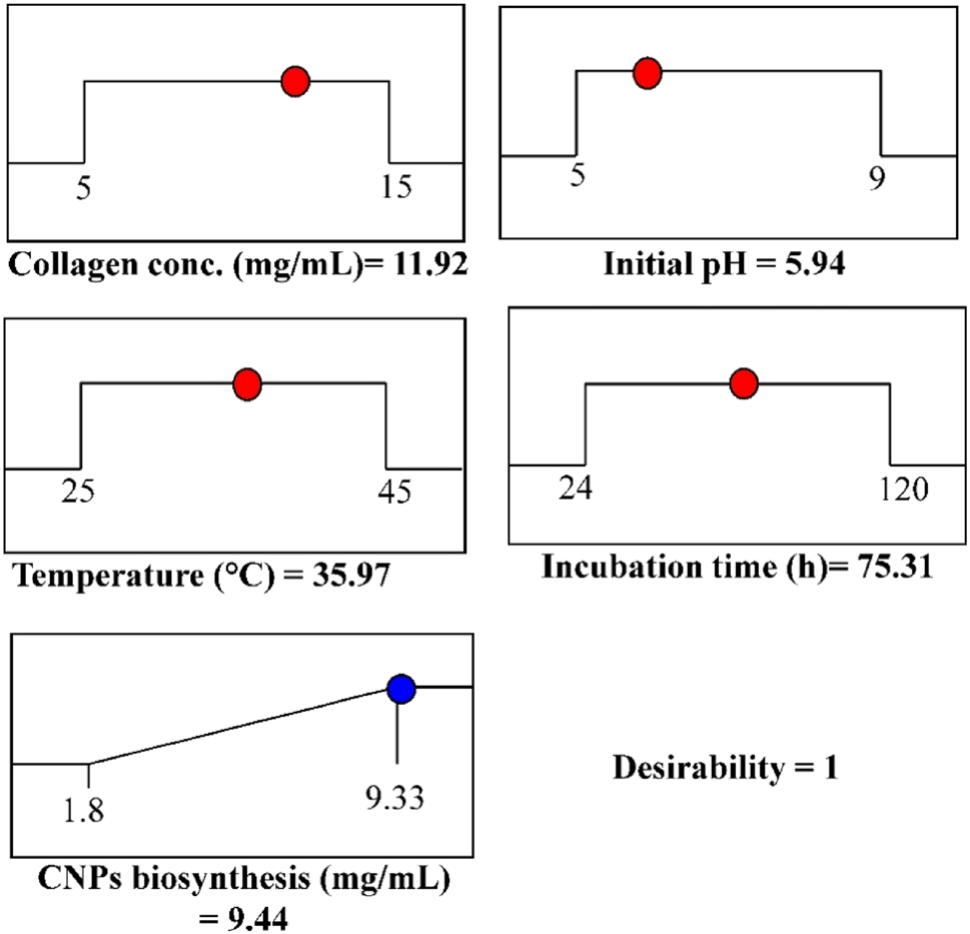
Optimization plotting reveals the optimum predicted values for maximum collagen- NPs biosynthesis by *Streptomyces plicatus* strain NEAA-3 and the desirability value. CNPs: collagen-NPs.

Desirability function analysis (DFA) is the most widely used technique for determining the optimal predicted conditions that would results in maximum of the response^80^. The values of the desirability functions range from 0 (undesirable) to 1(desirable). DFA was carried out by Design Expert Software (Version 12) in Fig. 11. The maximum theoretical yield of collagen-NPs biosynthesis using the cell-free supernatant of *Streptomyces plicatus* strain NEAA-3 was 9.44 mg/mL with a collagen concentration of 11.92 mg/mL, an initial pH of 5.94, an incubation time of 54.79 h, and a temperature of 35.79°C. The desirability function value reached 1. Under these conditions, collagen-NPs biosynthesis was practically verified. The collagen-NPs biosynthesis obtained experimentally was 9.53 mg/mL. The collagen-NPs biosynthesis value predicted by the polynomial model (9.44 mg/mL) was compared to the experimental result (9.53 mg/mL). A comparison was made between the collagen-NPs biosynthesis value predicted by the polynomial model (9.44 mg/mL) and the experimental result (9.53 mg/mL). The verification demonstrated a high degree of model accuracy, demonstrating the model's validity at the factor levels used in the current study. Using the desirability function approach, the collagen-NPs biosynthesis obtained after FCCD optimization (9.53 mg/mL) was 3.92 times more than the collagen-NPs biosynthesis obtained before optimization process (2.43 mg/mL).

### In vitro anticancer activity of collagen-NPs

**Figure 12:**
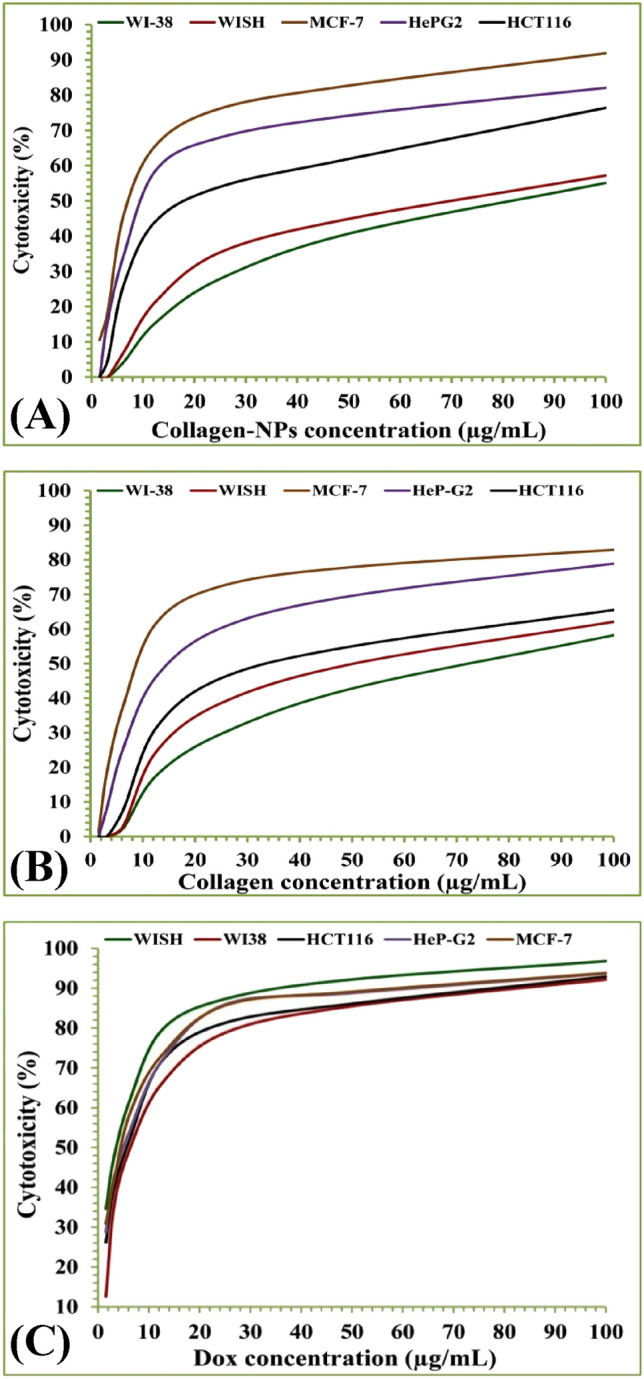
Diagram showing the anticancer (cytotoxicity) effect of collagen-NPs produced using *Streptomyces plicatus* strain NEAA-3 (**A**) and collagen (**B**) against different normal and cancer cell lines at different concentrations ranged from 1.65 to 100 µg. Doxorubicin used as standard anticancer drug.

In vitro tumor models are crucial tools for cancer studies and can be used as inexpensive screening platforms for medication therapy^94^. MTT assay is one of the most popular applications for assessing the cytotoxicity of various medications at various dosages. The MTT assay works on the basis that, in the majority of viable cells, mitochondrial activity. As a result, changes in mitochondrial activity are directly correlated with changes in the number of viable cells^95^. The MTT assay is based on the reduction of MTT, a yellow water-soluble tetrazolium dye, to purple formazan crystals, mostly by the action of mitochondrial dehydrogenases. The cytotoxic effects of collagen-NPs (Fig. 12A), collagen (Fig. 12B), and Dox, an available anticancer medication, (Fig. 11C), were examined in vitro using cancer cell lines (HCT116, HeP-G2 and MCF-7) and normal cell lines (WI38 and WISH). The inhibitory effect became apparent after 48 h of incubation. In comparison to untreated controls, the results are presented in Table 6 as growth inhibitory concentrations (IC_50_) values, or half-maximal inhibitory concentrations of cell growth after 48 h of incubation. The IC_50_ value of HCT116 was 24.82 ± 1.7 µg/mL (moderate inhibition), HeP-G2 was 13.36 ± 1.0 µg/mL (strong inhibition), and MCF-7 was 7.80 ± 0.5 µg/mL (strong inhibition) for collagen-NPs against cancer cell lines. Against normal cell lines, including WI38 and WISH, the IC_50_ of collagen-NPs was found to be 62.58 ± 3.6 and 74.91 ± 3.6 µg/mL, respectively. Therefore, the biosynthesized collagen-NPs showed a stronger cytotoxic effect on the cancer cell lines HCT116, HeP-G2, and MCF-7 compared to the normal cell lines WISH and WI38. Han et al.^96^ reported that the marine collagen reduced the growth of HepG2 and HeLa cells by 50% and 38%, respectively, with a concentration of 0.2 mg/mL. Collagen-NPs showed a higher cytotoxic effect than collagen. Studies on the size-dependent toxicity of micro- and nanoscale particles has demonstrated^97,98^ that the toxicity of nanoparticles is greater than that of bigger particles, supporting the theory that nanoparticles are generally more potent at causing damage. So, collagen-NPs could have a cytotoxic effect on mitochondrial, endoplasmic reticulum, and Golgi apparatus enzymes, as well as the lysosomal compartments, which reduce MTT dye to purple formazan.

**Table 6:** Showing the growth inhibitory concentration (IC_50_) values of collagen-NPs produced using *Streptomyces plicatus* strain NEAA-3, collagen, doxorubicin against different normal and cancer cell lines.

Component	In vitro cytotoxicity IC_50_ (µg)				
	WISH	WI38	HCT116	HeP-G2	MCF-7
DOX	3.34 ± 0.2	6.72 ± 0.5	5.23 ± 0.3	4.50 ± 0.2	4.17 ± 0.2
Collagen	53.25 ± 2.8	66.79 ± 3.3	41.67 ± 2.2	19.60 ± 1.2	11.62 ± 0.8
Collagen-NPs	74.91 ± 3.6	62.58 ± 3.1	24.82 ± 1.7	13.36 ± 1.0	7.80 ± 0.5

* IC_50_ (µg/mL): over 100 (non-cytotoxic), 51 – 100 (weak), 21 – 50 (moderate), 11 – 20 (strong) and 1–10 (very strong); DOX: Doxorubicin.

### In vivo apoptosis of EAC by collagen-NPs

**Figure 13:**
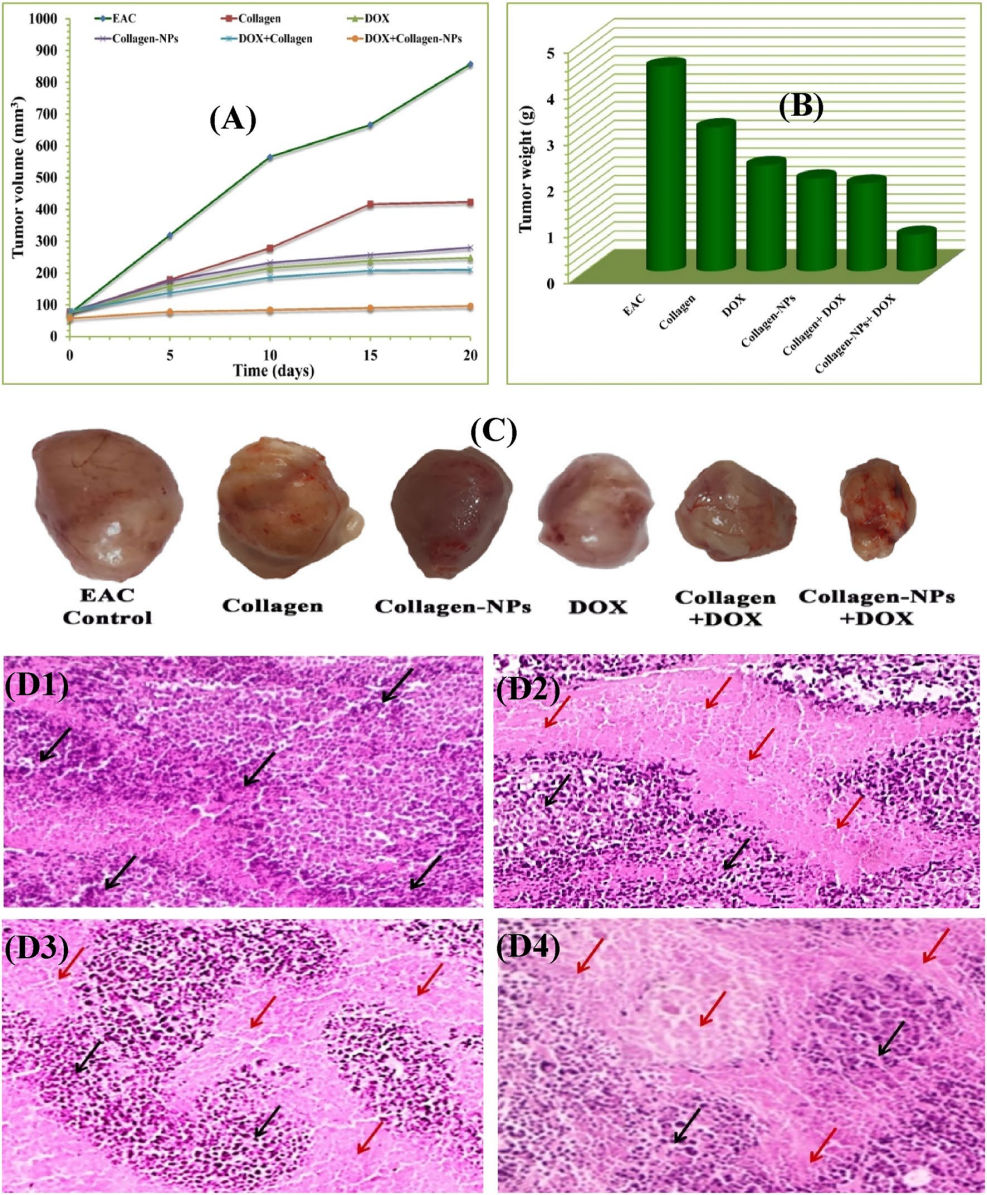
Effect of collagen-NPs by *Streptomyces plicatus* strain NEAA-3 and collagen curing alone or in integration with DOX on tumor volume (**A**) and tumor weight of EAC bearing mice (**B**); images of solid tumors at the same power of magnification, zooming and distance from camera (**C**); histopathological analysis micrographs on tumor sections of untreated mice bearing EAC (**D1**), mice bearing EAC cured with collagen-NPs (**D2**), mice bearing EAC cured with DOX (**D3**) and mice bearing EAC cured with collagen-NPs integrated with DOX (**D4**).

Certain chemical drugs may not always be able to cure cancer individually within in vivo cancer studies. Consequently, combining collagen-NPs in a synergistic approach with such a chemical drug is a promising way to increase efficiency. Collagen-based nanoparticles are thermally stable, reduce drug toxicity systemically, and enhance nanoparticle uptake by cells because of their biodegradability, biocompatibility, and mild antigenicity^8^. To evaluate the in vivo behavior of collagen-NPs as an efficient inducer of apoptosis, the effects of collagen-NPs and collagen-NPs/doxorubicin (DOX) in a synergistic combination therapy have been studied for their impact on the growth and death of solid tumors caused by Ehrlich ascites carcinoma (EAC) (Fig. 13, Table 7). The average tumor volume in EAC mice (the control group) increased from 72.86 to 857.84 mm^3^ after 20 days of treatment. Comparing tumor growth in EAC-bearing mice to EAC control animals, collagen, collagen-NPs, and DOX administration significantly slowed tumor growth by 55.89%, 74.24%, and 80.88%, respectively. Additionally, the mice treated with the collagen-NPs/DOX combination treatment showed significant tumor development inhibition (by 95.01%) when compared to animals treated with the DOX and collagen-NPs separately. In comparison to EAC control mice, collagen-NPs, DOX, and collagen-NPs/DOX were observed to significantly lower the weight of tumor lumps in mice. In contrast, mice getting the combined therapy had much lighter tumor lumps (0.79 ± 0.16) than mice receiving DOX (2.3 ± 0.31) or collagen-NPs (2.01 ± 0.37) injections.

**Table 7:** Suppression effect of collagen-NPs, collagen, DOX, collagen + DOX & collage-NPs + DOX on EAC bearing mice tumor parameters.

Groups	Tumor weight (g)	Tumor volume average (mm^3^)		ΔT/ΔC (%)	Inhibition (%)
		0 day	20 days		
EAC	4.44 ± 0.4	72.86	857.84	100.0	0.0
Collagen	3.12 ± 0.55	77.66	423.9	44.1	55.89
DOX	2.3 ± 0.31	75.2	225.28	19.12	80.88
Collagen-NPs	2.01 ± 0.37	78.14	280.36	25.76	74.24
Collagen + DOX	1.9 ± 0.05	80.6	210.44	24.53	75.47
Collagen-NPs + DOX	0.79 ± 0.16	57.64	96.88	4.99	95.01

The collagen used in the research is marine collagen (type I), which is totally safe as the LD_50_ of hydrolyzed collagen (30% solution in water; fish scale sourced; MW ~ 400 Da) was estimated to be greater than 2500 mg/kg body weight in Sprague–Dawley CD rats^99^**.** The acute toxicity test was conducted in compliance with OECD TG 423, the Organization for Economic Co-operation and Development's test guideline. According to Liang et al.^100^, the chronic toxicity assessment of MCP (marine collagen peptides) up to the diet concentration of 18%, estimated to be 8.586 g/kg body weight/day for females and 6.658 g/kg body weight/day for males, showed no evidence of a substantial detrimental effect or health risk. When given orally (up to 100%), hydrolyzed collagen was mostly harmless in acute toxicity tests involving mice and rats^101^. The test material was administered orally to three female rats in one group at a dose level of 2000 mg/kg body weight, and to three female rats in another group who had fasted, at the same dose level. During the 14 days following the dosage, no deaths or indications of systemic toxicity were noted. Every animal showed the anticipated increases in body weight. At necropsy, there were no abnormalities found^99^.

The anticancer activity of collagen is due to its amino acids. There are 19 amino acids (asparagine, threonine, serine, glutamic acid, alanine, valine, methionine, isoleucine, leucine, tyrosine, phenylalanine, histidine, lysine, and arginine) found in collagen, including hydroxyproline, that are unique to collagen and not found in other proteins. Its unusual amino acid makeup is distinguished by its high proline and glycine content as well as the lack of cysteine^102^. Glycine, lysine, and leucine are the main amino acid residues found in peptides with anticancer properties^103^. Hydrophobic positively charged lysine and arginine-rich peptides, for instance, function as cationic peptides and can interact with membranes through a snorkeling mechanism. This interaction can involve the selection of anionic membranes on cancer cells, the disruption of cell membrane integrity, penetration of the membrane, and possibly even a role in the toxicity of cancer cells^104^. Furthermore, histidine-containing peptides can cause cancer cytotoxicity through membrane permeability in acidic environments because of the protonation of histidine in acidic pH circumstances^105^. Remaining glutamic and aspartic acids may have anti-proliferative effects on tumor cells^106^.

Analysis of the histopathology of a tumor section stained with hematoxylin and eosin (Fig. 13) Massive malignant cells with anaplasia, pleomorphism, nuclear dyschromasia, multiple atypical nuclei, and condensed chromosomes were shown to proliferate quickly in mice with an untreated tumor (control A). These cells are depicted by the black arrows. When animals with EAC were treated with collagen-NPs (B) or DOX (C), the tumors' growth rates were slowed down, there were more necrotic areas (areas devoid of eosinophilic structures), there were noticeably more apoptotic bodies, and there was modest inhibition (blue arrows). A significant positive synergistic effect on the histopathological pattern was seen when collagen-NPs/DOX (D) were given to animals with EAC.

### Proposed mode of action

Either passive diffusion or non-specific permeation as a result of membrane damage caused by nanoparticles refers to the direct penetration of nanoparticles into cancer cells^107^. Through several mechanisms, ROS functions as a crucial signaling molecule to induce apoptosis. On one side, elevated lactate dehydrogenase (LDH) levels indicate that ROS cause cellular toxicity, such as damage to the cell membrane. Conversely, elevated ROS triggers the pro-inflammatory cytokine TNF-α, which in turn triggers p38 phosphorylation, caspase 8 and caspase 3 activation, and therefore initiates the apoptotic signaling cascade^108^. Other mechanisms include immunological interferences and transcription suppression. Collagen-NPs have a lethal effect on cancer cells as a result of the significant production (accumulation) of reactive oxygen species (ROS). The endoplasmic reticulum (ER), the mitochondria, and the peroxisomes are the places where ROS are produced most frequently^109^. Nanoparticles have been shown to interfere with the electron transport mechanism, which in turn causes an increase in the production of ROS within cells^110^, interference with mitochondrial function^111^, an increase in the ratio of NADP^+^ to NADPH. They also interfere with the expression of genes related to oxidative stress, such as *met9*, which is involved in NADPH production^112^, resulting in cell damage, lactate dehydrogenase (LDH) increasing and lipid peroxidation^113^, causing chromosome disintegration, aneuploid genic events, and DNA breaking (whether on single or double strands)^114,115^.

**Figure 14:**
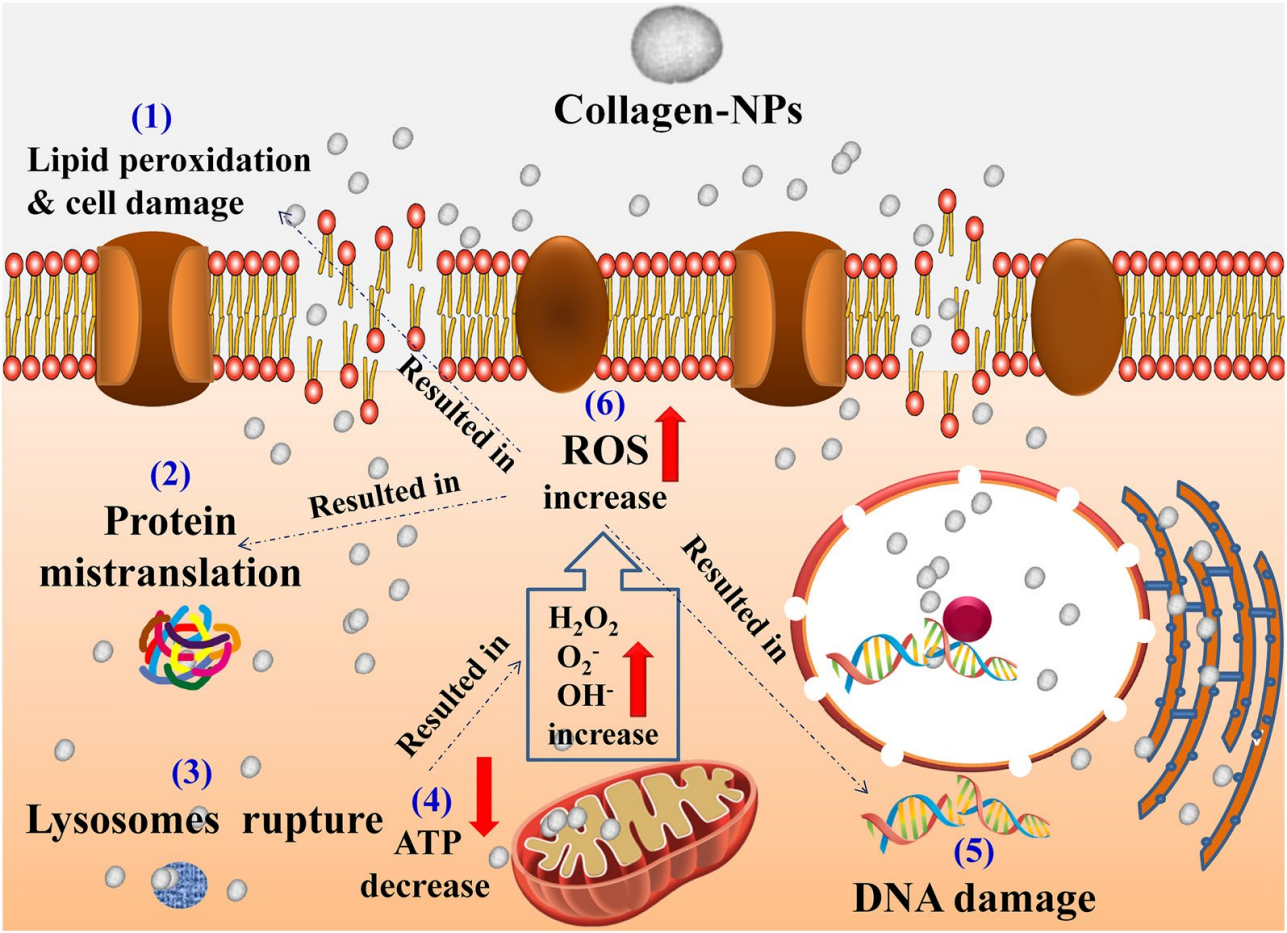
Schematic diagram showing the proposed mode of action of collagen-NPs produced by *Streptomyces plicatus* strain NEAA-3 against cancer cell.

Nanoparticles are particularly effective at inducing apoptosis due to their smaller size and better reactivity because of their higher surface area (Fig. 14). Therefore, the synergistic interaction between collagen-NPs and DOX might result in high amounts of (ROS) that may change the redox balance of the cell. Additionally, nanoparticles have been shown to increase protein damage, glutathione depletion, ATP depletion, and lysosome disintegration^116^. The majority of apoptotic effects manifest after tumor cells experience increased oxidative stress, which is followed by the release of inflammatory mediators that cause DNA and protein damage^117^. In response to oxidative stress, more proteins are structured to rearrange signaling and metabolic pathways. These modifications to the membrane and redox proteome alterations disturb the cycle of progress and proliferation, causing apoptosis and inhibiting malignancies^118^.

### Encapsulation efficiency and drug loading of MTX-loaded collagen-NPs

**Figure 15:**
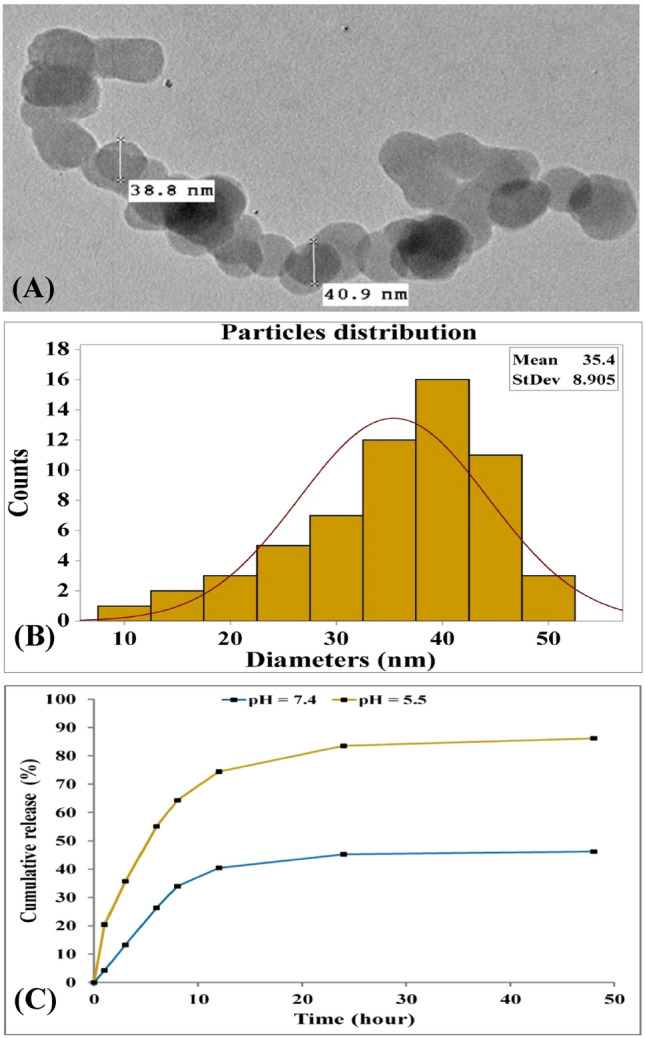
TEM image (**A**) of MTX loaded collagen-NPs, particles size distribution (**B**) and In vitro release of MTX loaded collagen-NPs (**C**).

Figure 15A displays the collagen-NPs that have been loaded with MTX and appear as spherical nanoparticles, as confirmed by the TEM image. The MTX-loaded collagen-NPs' diameter distribution was also examined. The histogram for the collagen-NPs that had been loaded with MTX is shown in Fig. 15B. The average size of the MTX-loaded collagen-NPs was about 35.4 ± 8.9 nm, which was larger than the previously estimated average size of collagen-NPs (33.15 ± 10.02). The encapsulation efficiency (EE) was 45.81%, and the drug loading (DL) was 22.67%. The majority of currently available nanomedicines do not have sufficient drug loading, usually less than 10%^119^. According to Musmade et al*.*^120^, the physicochemical characteristics of the MTX-loaded polymer (poly (D, L-lactide-coglycolide) (PLGA) nanoparticles were shown to be influenced by the drug-to-polymer ratio and stabilizer concentration. For every batch, the encapsulation efficiency percentage ranged from 8 to 16%. The difference in osmotic pressure between the two stages was the cause of the low encapsulation efficiency percentage.

### In vitro release of MTX-loaded collagen-NPs

The results demonstrated an initial burst release of the MTX, which was followed by an extended period of continuous release, according to the results (Fig. 15C). The percentage of MTX on the initial release may have contributed to its quick release from the nanoparticles' surface. The results of the in vitro drug release experiment showed that the biosynthesized collagen-NPs will have long-term drug release control. A brief initial release of 4.23% and 20.42% at pH 7.4 and 5.5, respectively, was followed by two phases of MTX release from NPs after one hour. At pH 7.4, the release rate after 12 h was 40.43% and 74.44%, respectively, which is considered to be a little high rate at this pH. Collagen-NPs may dissolve at pH 7.4 (collagen's isoelectric point), which may be the cause of this. The adsorbed MTX on the collagen-NPs' surface appeared to have a high rate of separation from the nanoparticles into the aqueous medium during the first 24 h (the CR% were 45.24% and 83.66% at pH 7.4 and 5.5, respectively). In contrast, at 48 h, the release rate was slower (the CR% were 46.31% and 86.52% at pH 7.4 and 5.5, respectively), due to the continuous hydrolysis and degradation of the collagen-NPs carried on by the penetration of the aqueous medium^121^. The MTX release rate of collagen-NPs that were loaded with MTX at a pH of 5.5 showed that the rate of MTX release increased as the pH decreased, and the highest value of MTX release occurred in the first 24 h. The cause of that result might be the instability of nanoparticles, which tend to assemble at an acidic pH^122^. Due to the highly acidic nature of tumor surfaces, this event shows that the medication was only effective against tumor tissue. Therefore, at lower pH levels, MTX can readily be released from nanoparticles in the medium and extracellular space of tumor cells. Collagen nanoparticles can efficiently penetrate tumor microenvironments and deliver anticancer therapies because collagen can resemble these environments^123^. The size, surface area, and absorption capacity of collagen nanoparticles are easily configurable; because of this, collagen nanoparticles are an excellent option for strategies involving the controlled release of medication^124^.

### Antioxidant efficiency using ABTS^+^ radical scavenging

Marine collagen is well-known for its antioxidant activity since it can shield skin cells from dangerous free radicals and oxidants that affect DNA, macromolecules, and cell membranes and speed the aging process of the skin^125^. The antioxidant capacities of collagen-NPs have been assessed by ABTS radical scavenging. The idea behind the ABTS approach is that antioxidants can eliminate cation colors (decolorization) by giving ABTS^+^ radicals a hydrogen atom. The findings showed that, in comparison to collagen, collagen-NPs have good antioxidant efficiency. The results showed that, in comparison to collagen (40.78%) and the activity of the common antioxidant ascorbic acid (89.6%), 500 µg/mL collagen-NPs demonstrated 60.39% radical scavenging effectiveness. Natural polymer collagen can be enzymatically hydrolyzed to generate collagen peptides with greater biological activity, which is crucial for preventing lipid oxidation, free radicals elimination, and maintaining the human body’s free radical balance, which lowers the danger of non-communicable chronic diseases^126^.

## Conclusion

In the current study, a novel and eco-friendly technique was described for the biosynthesis of collagen-NPs using *Streptomyces plicatus* strain NEAA-3. To the best of our knowledge, there has been no previous attempt has reported the biosynthesis of collagen-NPs using *Streptomyces plicatus*. All of the characterization analyses for collagen-NPs proved that the conversion of collagen to collagen-NPs by the cell-free supernatant of *Streptomyces plicatus* strain NEAA-3 as a bio-reductant agent is very promising. Optimization of the process variables was studied and the yield of collagen-NPs was maximized. The biosynthesized collagen-NPs using the cell-free supernatant of *Streptomyces plicatus* strain NEAA-3 have an average diameter of 33.15 ± 10.02 nm, making them promising for use in various pharmaceutical, and medical sectors. Moreover, both *in-vitro* and *in-vivo* investigations demonstrated the suitability of the biosynthesized collagen-NPs for a wide range of biomedical applications.

## Data Availability

All data generated or analyzed during this study are included in this article except the datasets that are available in the GenBank of The National Center for Biotechnology Information, (https://www.ncbi.nlm.nih.gov/nuccore/OR501412.1?report=GenBank).

## References

[1] DeFrates K, Markiewicz T, Gallo P (2018). Protein polymer-based nanoparticles: fabrication and medical applications. Int. J. Mol. Sci..

[2] Aditya A, Kim B, Koyani RD (2019). Kinetics of collagen microneedle drug delivery system. J. Drug Deliv. Sci. Technol..

[3] Kumar D (2013). A review on collagen based drug delivery systems. Int. J. Pharm. Teach. Pract..

[4] Xu S (2019). The role of collagen in cancer: From bench to bedside. J. Transl. Med..

[5] Lee JH (2018). Injectable hydrogels delivering therapeutic agents for disease treatment and tissue engineering. Biomater. Res..

[6] Shekhter AB, Fayzullin AL, Vukolova MN (2019). Medical applications of collagen and collagen-based materials. Curr. Med. Chem..

[7] Alarcon EI, Udekwu K, Skog M (2012). The biocompatibility and antibacterial properties of collagen-stabilized, photochemically prepared silver nanoparticles. Biomater.

[8] Arun A, Malrautu P, Laha A (2021). Collagen nanoparticles in drug delivery systems and tissue engineering. Appl. Sci..

[9] Przybyla DE, Chmielewski J (2010). Metal-triggered collagen peptide disk formation. J. Am. Chem. Soc..

[10] Jiang H, Liang G, Dai M (2020). Preparation of doxorubicin-loaded collagen-PAPBA nanoparticles and their anticancer efficacy in ovarian cancer. Ann. Transl. Med..

[11] Luo T, He L, Theato P, Kiick KL (2015). Thermoresponsive self-assembly of nanostructures from a collagen-like peptide-containing diblock copolymer. Macromol. Biosci..

[12] El-Naggar NEA, Abdelwahed NA, Darwesh OM (2014). Fabrication of biogenic antimicrobial silver nanoparticles by *Streptomyces aegyptia* NEAE 102 as eco-friendly nanofactory. J. Microbiol. Biotechnol..

[13] El-Naggar NEA, Abdelwahed NA (2014). Application of statistical experimental design for optimization of silver nanoparticles biosynthesis by a nanofactory *Streptomyces viridochromogenes*. J. Microbiol..

[14] Mohamedin A, El-Naggar NE, Shawqi Hamza S, Sherief AA (2015). Green synthesis, characterization and antimicrobial activities of silver nanoparticles by *Streptomyces viridodiastaticus* SSHH-1 as a living nanofactory: Statistical optimization of process variables. Curr. Nanosci..

[15] El-Naggar, N. E., Mohamedin, A., Hamza, S. S. & Sherief, A.-D. Extracellular biofabrication, characterization, and antimicrobial efficacy of silver nanoparticles loaded on cotton fabrics using newly isolated *Streptomyces* sp. SSHH-1E. *J. Nanomater. *(2016).

[16] El-Naggar NEA, Saber WI, Zweil AM, Bashir SI (2022). An innovative green synthesis approach of chitosan nanoparticles and their inhibitory activity against phytopathogenic *Botrytis cinerea* on strawberry leaves. Sci. Rep..

[17] El-Naggar NEA, Eltarahony M, Hafez EE (2023). (2023) Green fabrication of chitosan nanoparticles using Lavendula angustifolia, optimization, characterization and in-vitro antibiofilm activity. Sci Rep.

[18] El-Naggar NEA, Dalal SR, Zweil AM (2023). Artificial intelligence-based optimization for chitosan nanoparticles biosynthesis, characterization and in-vitro assessment of its anti-biofilm potentiality. Sci Rep.

[19] El-Naggar NE, Hussein MH, El-Sawah AA (2018). Phycobiliprotein-mediated synthesis of biogenic silver nanoparticles, characterization, *in vitro* and *in vivo* assessment of anticancer activities. Sci. Rep..

[20] El-Naggar NE, Hussein MH, El-Sawah AA (2017). Bio-fabrication of silver nanoparticles by phycocyanin, characterization, *in vitro* anticancer activity against breast cancer cell line and *in vivo* cytotxicity. Sci. Rep..

[21] El-Naggar NE-A, Hussein MH, Shaaban-Dessuuki SA, Dalal SR (2020). Production, extraction and characterization of *Chlorella vulgaris* soluble polysaccharides and their applications in AgNPs biosynthesis and biostimulation of plant growth. Sci. Rep..

[22] Bhainsa KC, D’souza SF (2006). Extracellular biosynthesis of silver nanoparticles using the fungus *Aspergillus fumigatus*. Colloids Surf. B.

[23] El-Naggar NEA, Rabei NH, Elmansy MF (2023). Artificial neural network approach for prediction of AuNPs biosynthesis by Streptomyces flavolimosus, characterization, antitumor potency in-vitro and in-vivo against Ehrlich ascites carcinoma. Sci. Rep..

[24] El-Naggar, N.E.A. Chapter 11—*Streptomyces*-based cell factories for production of biomolecules and bioactive metabolites, 183–234 (Academic Press, 2021).

[25] El-Naggar NE, Abdelwahed NA (2012). Optimization of process parameters for the production of alkali-tolerant carboxymethyl cellulase by newly isolated Streptomyces sp. strain NEAE-D. Afr. J. Biotechnol..

[26] El-Naggar NE (2015). Isolation, screening and identification of actinobacteria with uricase activity: Statistical optimization of fermentation conditions for improved production of uricase by *Streptomyces rochei* NEAE–25. Int. J. Pharmacol..

[27] El-Naggar NE, Hamouda R (2016). Antimicrobial potentialities of *Streptomyces lienomycini* NEAE-31 against human pathogen multidrug-resistant Pseudomonas aeruginosa. Int. J. Pharmacol..

[28] Shirling E, Gottlieb D (1966). Methods for characterization of *Streptomyces* species. Int. J. Syst. Evol. Microbiol..

[29] El-Naggar NE, Abdelwahed NA, Saber WI, Mohamed AA (2014). Bioprocessing of some agro-industrial residues for endoglucanase production by the new subsp.; Streptomyces albogriseolus subsp. cellulolyticus strain NEAE-J. Braz. J. Microbiol..

[30] Sambrook J, Fritsch EF, Maniatis T (1989). Molecular cloning.

[31] El-Naggar NEA, Sherief A, Hamza SS (2011). *Streptomyces aegyptia* NEAE 102, a novel cellulolytic streptomycete isolated from soil in Egypt. Afr. J. Microbiol. Res..

[32] Altschul SF, Madden TL, Schäffer AA (1997). Gapped BLAST and PSI-BLAST: A new generation of protein database search programs. Nucleic Acids Res..

[33] Saitou N, Nei M (1987). The neighbor-joining method: A new method for reconstructing phylogenetic trees. Mol. Biol. Evol..

[34] Mosmann T (1983). Rapid colorimetric assay for cellular growth and survival: application to proliferation and cytotoxicity assays. J. Immunol. Methods.

[35] Ozaslan M, Karagoz ID, Kilic IH (2011). Ehrlich ascites carcinoma. Afr. J. Biotechnol..

[36] El-Naggar NEA, Soliman HM, El-Shweihy NM (2018). Extracellular cholesterol oxidase production by *Streptomyces aegyptia*, in vitro anticancer activities against rhabdomyosarcoma, breast cancer cell-lines and in vivo apoptosis. Sci. Rep..

[37] Elsherbiny NM, Younis NN, Shaheen MA (2016). The synergistic effect between vanillin and doxorubicin in ehrlich ascites carcinoma solid tumor and MCF-7 human breast cancer cell line. Pathol. Res. Pract..

[38] Eisa NH, ElSherbiny NM, Shebl AM (2015). Phenethyl isothiocyanate potentiates anti-tumour effect of doxorubicin through Akt-dependent pathway. Cell Biochem. Funct..

[39] Singha HA, Sengupta M, Bawari M (2020). Neurobehavioral responses in swiss albino mice induced by an aqueous leaf extract from a medicinal plant named Heliotropium incanum Ruiz & Pav. Bioinformation.

[40] Sullivan PM, Reed SJ, Kalia V (2021). Solid tumor microenvironment can harbor and support functional properties of memory T cells. Front. Immunol..

[41] Schirner M, Hofmann J, Menrad A, Schneider MR (1998). Antiangiogenic chemotherapeutic agents: Characterization in comparison to their tumor growth inhibition in human renal cell carcinoma models. Clin. Cancer Res..

[42] Zhao XQ, Li WJ, Jiao WC (2009). *Streptomyces*
*xinghaiensis* sp. Nov., isolated from marine sediment. Int. J. Syst. Evol. Microbiol..

[43] Goodfellow M, Kämpfer P, Busse H-J (2012). Bergey’s manual of systematic bacteriology.

[44] Shoge M, Amusan T (2020). J. Biotechnol..

[45] Fratianni F, d’Acierno A, Ombra MN (2021). Fatty acid composition, antioxidant, and *in vitro* anti-inflammatory activity of five cold-pressed prunus seed oils, and their anti-biofilm effect against pathogenic bacteria. Front. Nutr..

[46] Belwal T, Nabavi SM, Nabavi SF (2021). Naturally occurring chemicals against Alzheimer's disease. AP.

[47] Narang D, Sood S, Thomas MK (2004). Effect of dietary palm olein oil on oxidative stress associated with ischemicreperfusion injury in isolated rat heart. BMC Pharmacol..

[48] Kulkarn (2015). Methane sulphonic acid is green catalyst in organic synthesis. Orient. J. Chem..

[49] Singh AN, Yethiraj A (2021). Liquid–liquid phase separation as the second step of complex coacervation. J. Phys. Chem. B.

[50] Nagaraja U, Kawakami K, Zhang S (2014). Fabrication of solid collagen nanoparticles using electrospray deposition. Chem. Pharm. Bull..

[51] Cinar S, Gündül G, Mavis B, Colak U (2011). Synthesis of silver nanoparticles by oleylamine-oleic acid reduction and its use in making nanocable by coaxial electrospinning. J. Nanosci. Nanotechnol..

[52] Gupta R, Pancholi K, De Sa R (2019). Effect of oleic acid coating of iron oxide nanoparticles on properties of magnetic polyamide-6 nanocomposite. JOM.

[53] Xia Y, Xie M, Lyu Z, Chen R (2020). Mechanistic study of the multiple roles of oleic acid in the oil phase synthesis of Pt nanocrystals. Chem. Eur. J..

[54] Mourdikoudis S, Menelaou M, Fiuza-Maneiro N (2022). Oleic acid/oleylamine ligand pair: A versatile combination in the synthesis of colloidal nanoparticles. Nanoscale Horiz.

[55] Agrawal N, Munjal S, Ansari MZ, Khare N (2017). Superhydrophobic palmitic acid modified ZnO nanoparticles. Ceram Int..

[56] Saallah S, Roslan J, Julius FS (2021). Comparative study of the yield and physicochemical properties of collagen from sea cucumber (*Holothuria scabra*), obtained through dialysis and the ultrafiltration membrane. Mol.

[57] Yan M, Li B, Zhao X (2008). Characterization of acid-soluble collagen from the skin of walleye pollock (*Theragra chalcogramma*). Food Chem..

[58] Adibzadeh N, Aminzadeh S, Jamili S (2014). Purification and characterization of pepsin-solubilized collagen from skin of sea cucumber *Holothuria parva*. Appl. Biochem. Biotechnol..

[59] Naskar A, Kim KS (2020). Recent advances in nanomaterial-based wound-healing therapeutics. Pharmaceutics.

[60] Kong J, Yu S (2007). Fourier transform infrared spectroscopic analysis of protein secondary structures. Acta Biochim. Biophys. Sin..

[61] Pati F, Adhikari B, Dhara S (2010). Isolation and characterization of fish scale collagen of higher thermal stability. Bioresour. Technol..

[62] Movasaghi Z, Rehman S, Rehman I (2008). Fourier transform Infrared (FTIR) spectroscopy of biological tissues. Appl. Spect. Rev..

[63] Haris PI, Severcan F (1999). FTIR spectroscopic characterization of protein structure in aqueous and non-aqueous media. J. Mol. Catal. B-Enzym..

[64] Lambert JB (1987). introduction to organic spectroscopy.

[65] Faralizadeh S, Rahimabadi EZ, Bahrami SH (2021). Extraction, characterization and biocompatibility evaluation of collagen from silver carp (*Hypophthalmichthys molitrix*) skin by-product. Sustain. Chem. Pharm..

[66] Liu A, Zhaohui Z, Hou H (2018). Characterization of acid- and pepsin-soluble collagens from the cuticle of *Perinereis nuntia* (Savigny). Food Biophys..

[67] Cameron GJ, Cairns DE, Wess TJ (2007). The variability in type I collagen helical pitch is reflected in the D periodic fibrillar structure. J. Mol. Biol..

[68] Sharma D, Maheshwari D, Philip G (2014). Formulation and optimization of polymeric nanoparticles for intranasal delivery of lorazepam using Box-Behnken design: In vitro and in vivo evaluation. Biomed. Res. Int..

[69] Khan I, Saeed K, Khan I (2019). Nanoparticles: Properties, applications and toxicities. Arab. J. Chem..

[70] El-Sawah AA, El-Naggar NE, Eldegla HE, Soliman HM (2024). Green synthesis of collagen nanoparticles by *Streptomyces xinghaiensis* NEAA-1, statistical optimization, characterization, and evaluation of their anticancer potential. Sci. Rep..

[71] Charles J, Ramkumaar GR, Azhagiri S (2009). FTIR and thermal studies on nylon-66 and 30% glass fibre reinforced nylon-66. E-J. Chem..

[72] Ebnesajjad S (2006). 4 - Surface and material characterization techniques. Surf. Treat. Mater. Adhesion Bond..

[73] Lohrasbi S, Mirzaei E, Karimizade A (2020). Collagen/cellulose nanofiber hydrogel scaffold: physical, mechanical and cell biocompatibility properties. Cellulose.

[74] Tampieri A, Celotti G, Landi E (2003). Biologically inspired synthesis of bone-like composite: Self-assembled collagen fibers/hydroxyapatite nanocrystals. J. Biomed. Mater. Res. A.

[75] Batista MP, Fernández N, Gaspar FB (2022). Extraction of biocompatible collagen from blue shark skins through the conventional extraction process intensification using natural deep eutectic solvents. Front. Chem..

[76] Protasevich I, Ranjbar B, Lobachov V (1997). Conformation and thermal denaturation of apocalmodulin: Role of electrostatic mutations. Biochemistry.

[77] Jaziri AA, Shapawi R, Mohd Mokhtar RA (2022). Biochemical analysis of collagens from the bone of lizardfish (Saurida tumbil Bloch, 1795) extracted with different acids. Peer J.

[78] El-Naggar NEA, El-khateeb AY, Ghoniem AA (2020). Saber, Innovative low-cost biosorption process of Cr^6+^ by *Pseudomonas alcaliphila* NEWG-2. Sci. Rep..

[79] El-Naggar NEA, El-Shweihy NM, El-Ewasy SM (2016). Identification and statistical optimization of fermentation conditions for a newly isolated extracellular cholesterol oxidase-producing *Streptomyces cavourensis* strain NEAE-42. BioMed Central Microbiol..

[80] El-Naggar NEA, Hamouda RA, Saddiq AA, Alkinani MH (2021). Simultaneous bioremediation of cationic copper ions and anionic methyl orange azo dye by brown marine alga *Fucus vesiculosus*. Sci Rep.

[81] El-Naggar NEA, El-Shweihy NM (2020). Bioprocess development for L-asparaginase production by *Streptomyces rochei*, purification and *in-vitro* efficacy against various human carcinoma cell lines. Sci Rep.

[82] Hamouda RA, El-Naggar NE, Doleib NM, Saddiq AA (2020). Bioprocessing strategies for cost-effective simultaneous removal of chromium and malachite green by marine alga *Enteromorpha intestinalis*. Sci. Rep..

[83] El-Naggar NEA, Hamouda RA, El-Khateeb AY, Rabei NH (2021). Biosorption of cationic Hg^2+^ and Remazol brilliant blue anionic dye from binary solution using *Gelidium corneum* biomass. Sci Rep.

[84] Samuel, E.A. & Oladipupo, O.O. Factorial designs application to study enhanced bioremediation of soil artificially contaminated with weathered bonny light crude oil through biostimulation and bioaugmentation strategy. *JEP* **3**, (2012).

[85] Ibrahim AM, Hamouda RA, El-Naggar NEA (2021). Bioprocess development for enhanced endoglucanase production by newly isolated bacteria, purification, characterization and in-vitro efficacy as anti-biofilm of Pseudomonas aeruginosa. Sci Rep.

[86] El-Naggar NEA, Moawad H, Abdelwahed NA (2017). Optimization of fermentation conditions for enhancing extracellular production of L-asparaginase, an anti-leukemic agent, by newly isolated *Streptomyces brollosae* NEAE-115 using solid state fermentation. Ann. Microbiol..

[87] Ghoniem AA, El-Naggar NEA, Saber WIA (2020). Statistical modeling-approach for optimization of Cu^2+^ biosorption by *Azotobacter*
*nigricans* NEWG-1; characterization and application of immobilized cells for metal removal. Sci. Rep..

[88] El-Naggar NEA, Moawad H, El-Shweihy NM (2019). Process development for scale-up production of a therapeutic L-asparaginase by Streptomyces brollosae NEAE-115 from shake flasks to bioreactor. Sci. Rep..

[89] El-Naggar NEA, Haroun SA, El-Weshy EM (2019). Mathematical modeling for bioprocess optimization of a protein drug, uricase, production by *Aspergillus welwitschiae* strain 1–4. Sci. Rep..

[90] Yan M, Li B, Zhao X, Qin S (2012). Effect of concentration, pH and ionic strength on the kinetic self-assembly of acid-soluble collagen from walleye pollock (Theragra chalcogramma) skin. Food Hydrocoll..

[91] Li Y, Asadi A, Monroe MR, Douglas EP (2009). PH effects on collagen *fibrillogenesis in vitro*: electrostatic interactions and phosphate binding. Mat. Sci. Eng. C.

[92] Song X, Wang Z, Tao S *et al*. Observing effects of calcium/magnesium ions and pH value on the self-assembly of extracted swine tendon collagen by atomic force microscopy. *J. Food. Sci.* (2017) 8

[93] Marelli B, Ghezzi CE, Zhang YL (2015). Fibril formation pH controls intrafibrillar collagen biomineralization *in vitro* and *in vivo*. Biomater.

[94] Katt ME, Placone AL, Wong AD (2016). In vitro tumor models: advantages, disadvantages, variables, and selecting the right platform. Front. Bioeng. Biotechnol..

[95] Meerloo JV, Kaspers GJ, Cloos J (2011). Cell sensitivity assays: the MTT assay. Methods Mol. Biol..

[96] Han SH, Uzawa Y, Moriyama T (2011). Effect of collagen and collagen peptides from Bluefin tuna abdominal skin on cancer cells. Health.

[97] Brown DM, Wilson MR, MacNee W (2001). Size-dependent proinflammatory effects of ultrafine polystyrene particles: a role for surface area and oxidative stress in the enhanced activity of ultrafnes. Toxicol. Appl. Pharmacol..

[98] Li Y, Sun L, Jin M (2011). Size-dependent cytotoxicity of amorphous silica nanoparticles in human hepatoma HepG2 cells. Toxicol. in Vitro.

[99] Burnett CL, Bergfeld WF, Belsito DV (2022). Safety assessment of skin and connective tissue-derived proteins and peptides as used in cosmetics. Int. J. Toxicol..

[100] Liang J, Pei XR, Zhang ZF (2012). A chronic oral toxicity study of marine collagen peptides preparation from chum salmon (Oncorhynchus keta) skin using Sprague-Dawley rat. Mar. Drugs.

[101] Elder RL (1985). Final report on the safety assessment of hydrolyzed collagen. JACT.

[102] Gauza-Włodarczyk M, Kubisz L, Włodarczyk D (2017). Amino acid composition in determination of collagen origin and assessment of physical factors effects. Int. J. Biol. Macromol..

[103] Shoombuatong W, Schaduangrat N, Nantasenamat C (2018). Unraveling the bioactivity of anticancer peptides as deduced from machine learning. Excli. J..

[104] Dai YX, Cai XG, Shi W (2017). Pro-apoptotic cationic host defense peptides rich in lysine or arginine to reverse drug resistance by disrupting tumor cell membrane. Amino Acids.

[105] Navarro S, Aleu J, Jimenez M (2008). The cytotoxicity of eosinophil cationic protein/ribonuclease 3 on eukaryotic cell lines takes place through its aggregation on the cell membrane. Cell Mol. Life Sci..

[106] Yamaguchi Y, Yamamoto K, Sato Y (2016). Combination of aspartic acid and glutamic acid inhibits tumor cell proliferation. Biomed. Res..

[107] Bartlomiejczyk T, Lankoff A, Kruszewski M, Szumiel I (2013). Silver nanoparticles-allies or adversaries?. Ann. Agr. Environ. Med..

[108] Ma DD, Yang WX (2016). Engineered nanoparticles induce cell apoptosis: potential for cancer therapy. Oncotarget..

[109] Murphy MP (2009). How mitochondria produce reactive oxygen species. Biochem. J..

[110] Wang G, Jin W, Qasim AM (2017). Antibacterial effects of titanium embedded with silver nanoparticles based on electron-transfer-induced reactive oxygen species. Biomater.

[111] AshaRani PV, Low Kah Mun G, Hande MP (2009). Cytotoxicity and genotoxicity of silver nanoparticles in human cells. ACS Nano..

[112] Zhang Y, Gu AZ, Xie S (2018). Nano-metal oxides induce antimicrobial resistance via radical-mediated mutagenesis. Environ. Int..

[113] Gu L, Li Q, Quan X (2014). Comparison of nanosilver removal by flocculent and granular sludge and short- and long-term inhibition impacts. Water Res..

[114] Kang SJ, Kim BM, Lee YJ, Chung HW (2008). Titanium dioxide nanoparticles trigger p53-mediated damage response in peripheral blood lymphocytes. Environ. Mol. Mutagen.

[115] Di Bucchianico S, Fabbrizi MR, Cirillo S (2014). Aneuploidogenic effects and DNA oxidation induced in vitro by differently sized gold nanoparticles. Int. J. Nanomed..

[116] Canesi L, Ciacci C, Fabbri R (2012). Bivalve molluscs as a unique target group for nanoparticle toxicity. Mar. Environ. Res..

[117] Raj S, Khurana S, Choudhari R (2021). Specific targeting cancer cells with nanoparticles and drug delivery in cancer therapy. Semin. Cancer Biol..

[118] López Grueso MJ, Tarradas Valero RM, Carmona-Hidalgo B (2019). Peroxiredoxin 6 down-regulation induces metabolic remodeling and cell cycle arrest in HepG2 cells. Antioxidants.

[119] Shen S, Wu Y, Liu Y, Wu D (2017). High drug-loading nanomedicines: Progress, current status, and prospects. Int. J. Nanomed..

[120] Musmade KP, Deshpande PB, Musmade PB (2014). Methotrexate-loaded biodegradable nanoparticles: Preparation, characterization and evaluation of its cytotoxic potential against U-343 MGa human neuronal glioblastoma cells. Bull. Mater. Sci..

[121] Maleki H, Dorkoosh F, Adabi M (2017). Methotrexate-loaded PLGA nanopartcles: preparaton, characterizaton and their cytotoxicity effect on human glioblastoma U87MG cells. Int. J. Med. Nano Res..

[122] Du S, Kendall K, Toloueinia P (2012). Aggregation and adhesion of gold nanoparticles in phosphate bufered saline. J. Nanopart. Res..

[123] Le V-M, Lang M-D, Shi W-B (2016). A collagen-based multicellular tumor spheroid model for evaluation of the efficiency of nanoparticle drug delivery. Artif. Cells Nanomed. Biotechnol..

[124] Nitta SK, Numata K (2013). Biopolymer-based nanoparticles for drug/gene delivery and tissue engineering. Int. J. Mol. Sci..

[125] Carvalho, D. N., Inácio, A. R. & Sousa, R. O. *et al.* Seaweed polysaccharides as sustainable building blocks for biomaterials in tissue engineering in sustainable seaweed technologies. *Elsevier* 543–587 (2020b).

[126] Jideani AIO (2021). Antioxidant-rich natural fruit and vegetable products and human health. Int. J. Food Prop..

